# Surface Reconstruction Facilitated by Fluorine Migration and Bimetallic Center in NiCo Bimetallic Fluoride Toward Oxygen Evolution Reaction

**DOI:** 10.1002/advs.202306758

**Published:** 2023-12-03

**Authors:** Zhenhang Xu, Wei Zuo, Yueying Yu, Jinyan Liu, Gongzhen Cheng, Pingping Zhao

**Affiliations:** ^1^ College of Chemistry and Molecular Sciences Wuhan University Wuhan Hubei 430072 P. R. China; ^2^ School of Nursing Wuhan University Wuhan Hubei 430072 P. R. China; ^3^ Department of Biological and Chemical Engineering Zhixing College of Hubei University Wuhan Hubei 430011 P. R. China

**Keywords:** bimetallic fluoride, fluorine migration, oxygen evolution reaction, surface reconstruction

## Abstract

Oxygen evolution reaction (OER) is a critical anodic reaction of electrochemical water splitting, developing a high‐efficiency electrocatalyst is essential. Transition metal‐based catalysts are much more cost‐effective if comparable activities can be achieved. Among them, fluorides are rarely reported due to their low aqueous stability of coordination and low electric conductivity. Herein, a NiCo bimetallic fluoride with good crystallinity is designed and constructed, and significantly enhanced catalytic activity and conductivity are observed. The inevitable oxidation of transition metal ions at high potential and the dissociation of F^−^ are attributed to the low aqueous stability of coordination. The theoretical researches predicte that transition metal fluorides should have a strong tendency to electrochemical reconstruction. Therefore, based on the observations on their electrochemical behavior, high‐resolution transmission electron microscopy, X‐ray photoelectron spectroscopy, and bode plots, it is further demonstrated that surface reconstruction occurred during the electrochemical process, meanwhile a significant increase of electrochemically active area, which is created by F migration, are also directly observed. Additionally, DFT calculation results show that the electronic structure of the catalysts is modulated by the bimetallic centers, and this reconstruction helps optimizing the adsorption energy of oxygen‐containing species and improves OER activity.

## Graphical abstract

Novel solid–liquid contact modification and one‐step vapor phase fluorination strategies are adopted to synthesize stable and efficient NiCo bimetallic fluoride Ni_0.42_Co_0.58_F_2_‐G with high crystallinity, which exhibited a significant enlarged ECSA by F migration during surface reconstruction. DFT calculations indicate that bimetallic center can induce redistribution of electrons and optimize the adsorption of intermediates, further facilitating surface reconfiguration and OER kinetics.

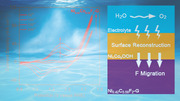

## 1 Introduction

Fossil energy sources are becoming increasingly depleted, researchers worldwide are actively seeking alternatives to alleviate this situation. Among a range of renewable resources, hydrogen energy, with its reaction by‐product of water,^[^ ^1^ ^]^ is regarded as one of the best due to its stability and reliability compared to wind and solar energy Electrochemical water splitting is an attractive pathway that can conveniently achieve the conversion from electrical to hydrogen energy.^[^ ^2^ ^]^ However, the anionic half‐reaction, OER requires a much larger overpotential compared with the cathodic HER due to the multi‐step proton/electron coupling, which undoubtedly hinders the efficient conversion of energy.^[^ ^3^ ^]^ Which means that the OER needs to be driven during the electrocatalytic process, The volcano‐type relationship between the adsorption energies of different oxygen‐containing intermediates on the active sites can also provide insights to helping the investigation of the efficient OER catalysts. Among them, catalysts such as RuO_2_ and IrO_2_ are situated in the high position of volcano chart due to their optimized adsorption relationships, their superior electrochemical performance.^[4]^have also been observed. Unfortunately, the expensive price and scarce reserves severely limit their large‐scale industrial application. Alternative solutions, such as reducing the amount of precious metals or using cheaper electrocatalysts, have been developed and great achievements have been made.^[^ ^5^ ^]^

Among various alternatives, transition metal‐based electrocatalysts have been widely examined for their unparalleled advantages such as inexpensiveness and abundance on earth.^[^ ^6^ ^]^ A large quantity of transition metal‐based carbides,^[^ ^7^ ^]^ nitrides,^[^ ^8^ ^]^ oxides,^[^ ^9^ ^]^ hydroxides,^[^ ^10^ ^]^ phosphides^[^ ^11^ ^]^ and sulfides^[^ ^12^ ^]^ have been studied for efficient OER. It is noteworthy that fluorides have been reported as electrocatalysts much less frequently than the transition metal‐based derivatives mentioned above, those fluorides reported are mostly monometallic fluorides such as CoF_2_,^[^ ^13^ ^]^ or fluoride perovskite.^[^ ^14^ ^]^ The reason behind may be due to the low conductivity caused by their low aqueous stability of coordination between metal and fluoride in alkaline electrolytes.^[^ ^13^ ^]^ However, fluorides have their unique benefits, the most outstanding advantage is that fluorine has the largest electronegativity, which can push the valence of the metal centers to the highest. The induction effects of high‐valent metal centers can assist the surface reconstruction, forming amorphous structure and so on. Consequently, fluorides are the potent catalyst for oxygen evolution reaction. Recent studies have demonstrated that at high potentials of OER, most transition metal‐based derived electrocatalysts were prone to surface reconstruction, whose products are likely to play the role of actual active sites in the reaction process, and catalysts with such properties are called pre‐catalysts.^[^ ^15^ ^]^ Compared with the heterostructure catalysts synthesized by general methods, the heterostructures formed during the electrochemical activation of the pre‐catalysts combined with the substrate could have better electrocatalytic performance, which may be attributed to the electronic structure optimization or the exposure of more active sites.^[^ ^16^ ^]^

Herein, we report a novel NiCo bimetallic fluoride for efficient water oxidation and provide an insight into its surface reconfiguration phenomenon during electrochemical processes. NiCo‐based hydroxide nanoarrays with extremely high specific surface area were first fabricated by a novel solid‐liquid contact modification method. Subsequent transformation into NiCo bimetallic fluoride via vapor phase fluorination in one step. As a pre‐catalyst, the NiCo bimetallic fluoride Ni_0.42_Co_0.58_F_2_‐G exhibited excellent electrochemical activity compared to the monometallic‐based fluoride after complete electrochemical activation. XPS analysis showed that F migrated during the electrochemical surface reconstruction process, exposing more electrochemically active sites. From the Bode plots, it is clear that the presence of bimetallic center can help in optimizing the electronic structure and thus facilitating surface reconstruction. DFT results suggested that the construction of heterostructures as well as the presence of bimetallic center assist very well in optimizing the OER reaction steps and boosting the electrochemical process.

## 2 Results and Discussion

### 2.1 Construction and Characterization of NiCo Bimetallic Fluoride Ni_0.42_Co_0.58_F_2_‐G

**Figure 1:**
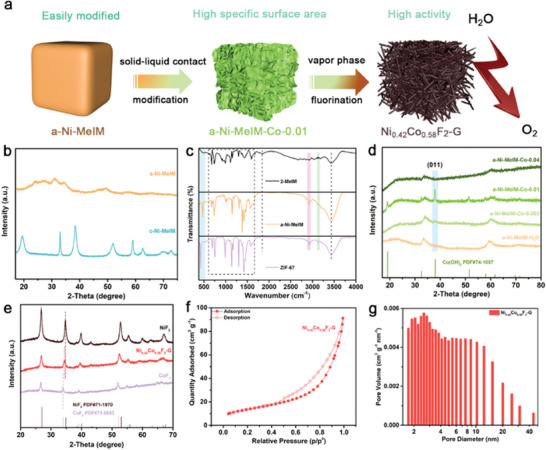
Phase and bonding structure of studied samples. a) Schematic diagram for the generation process of Ni_0.42_Co_0.58_F_2_‐G; b) XRD patterns of a‐Ni‐MeIM and c‐Ni‐MeIM; c) FTIR spectra of 2‐MeIM, a‐Ni‐MeIM and ZIF‐67; d) XRD patterns of a‐Ni‐MeIM treated by different concentration of Co^2+^; e) XRD patterns of different fluoride; f) N_2_ adsorption–desorption isotherms and g) pore size distribution curves of Ni_0.42_Co_0.58_F_2_‐G.

As depicted in **Figure** **1**a, Ni_0.42_Co_0.58_F_2_‐G was synthesized from pre‐constructed NiCo‐based nanoarray precursor by a facile one‐step vapor phase fluorination method described in the experimental section. First of all, a‐Ni‐MeIM, a type of metal‐imidazole‐based complex with low crystallinity was devised and prepared, which exhibited amorphous in shape (Figure S1, Supporting Information). X‐ray diffraction (XRD) pattern of a‐Ni‐MeIM showed weak broad peaks and no obvious characteristic sharp peaks were observed, as shown in Figure 1b (the orange line), indicating amorphous properties. In order to further obtain the functional group and bonding information of a‐Ni‐MeIM, Fourier Transform Infrared (FTIR) spectra of 2‐methylimidazole based complexes including a‐Ni‐MeIM and ZIF‐67 were recorded. It is obvious that both of a‐Ni‐MeIM and 2‐methylimidazole displayed analogous characteristic absorption at 600–1600 cm^−1^, which can be assigned to the characteristic peaks of imidazole ring, as exhibited in Figure 1c. The broad peaks located at ≈3430 cm^−1^ can be attributed to the O─H stretching vibration, the peaks located at 2925 and 3133 cm^−1^ were corresponding to the stretching vibration mode of C─H on the imidazole ring and methyl group, respectively.^[^ ^17^ ^]^ Interestingly, the N─H stretching vibration peaks on the imidazole ring located at 1843 cm^−1^ disappeared and a new signal showed stronger spikes at 473 cm^−1^, corresponding to the stretching vibration peaks of Ni–N,^[^ ^18^ ^]^ in a‐Ni‐MeIM. Similar phenomenon that the stretching vibration of Co─N occurred at 425 cm^−1^ was also observed in the complex ZIF‐67, synthesized by Co^2+^ and 2‐methylimidazole. Under the same experimental conditions, using a larger ratio of nickel nitrate and 2‐methylimidazole as the precursor will result in a complex c‐Ni MeIM with higher crystallinity. Different from a‐Ni‐MeIM, XRD Pattern of c‐Ni‐MeIM exhibited a set of highly characteristic crystalline peaks, shown in Figure 1b (the blue line).^[^ ^19^ ^]^ The synthesis process of a‐Ni‐MeIM and c‐Ni‐MeIM was compared, it was found that a much larger concentration of ligand in the precursor solution assists the generation of a larger number of short‐chain complexes, leading to the formation of a‐Ni‐MeIM; while a lower concentration of ligand would help in forming stable long‐chain structure growth, leading to the formation of c‐Ni‐MeIM. This is further validated with infrared spectroscopy, the IR spectrum of c‐Ni‐MeIM showed significantly different signals to that of a‐Ni‐MeIM (Figure S2, Supporting Information). A broad strong absorption band located at ≈500 cm^−1^ indicates that a large number of Ni–N coordination structures are present in c‐Ni‐MeIM, and thus a long chain complex of c‐Ni‐MeIM can be determined. However, a‐Ni‐MeIM showed characteristics of short chain complex, which facilitates the subsequent modification to creating NiCo‐based nanoarray precursor and its fluorides with high electrochemical activity (vide infra).

To further improve the structure of a‐Ni‐MeIM, Co^2+^ was introduced into this system. It was found that structure of the product a‐Ni‐MeIM‐Co‐α relied highly on the Co^2+^ concentration (*α* indicates the concentration of Co^2+^ used). a‐Ni‐MeIM‐Co‐0.01 exhibited a set of obvious peaks of Co(OH)_2_ after Co^2+^ hydrolysis treatment_,_ as shown in Figure 1d. At the same time, typical layered structure of Co(OH)_2_ can be observed in a‐Ni‐MeIM ‐Co‐0.01, as shown in Figure S3 (Supporting Information). Furthermore, concentration gradient method was utilized to reveal the evolution of hydrolysis process (Figure S4, Supporting Information). The intensity of the peak corresponds to (011) crystal facet of Co(OH)_2_ in Figure 1d gradually increased with the increment of Co^2+^ concentration from zero, which demonstrated that a‐Ni‐MeIM was partially dissolved by H^+^ due to its weak crystallinity, during which Ni^2+^ was moderately dissoluted into solution while Co^2+^ entered the lattice and generated Co(OH)_2_ nanoarrays due to the strong coordination of Co^2+^ with N. It is noted that a‐Ni‐MeIM‐Co‐0.04 showed an amorphous character with no obvious peaks of Co(OH)_2_ observed. One possible reason is that the hydrolysis rate of a‐Ni‐MeIM and the nucleation rate of Co(OH)_2_ were too drastic to form stable crystalline form under large concentration of Co^2+^, which was also confirmed by the concentration of Ni^2+^ and Co^2+^ in the supernatant after treatment with different Co^2+^ concentrations (Table S2, Supporting Information). From Table S2, it can be seen that under the condition of 0.04 m Co^2+^, a large amount of Ni was leached out from a‐Ni‐MeIM in the supernatant, indicating that the rapid hydrolysis reaction of Co^2+^ will lead to such result. Up to this point, NiCo‐based nanoarray precursor with a significantly higher specific surface compared to a‐Ni‐MeIM was prepared by a concise solid–liquid exchange method, which facilitated the construction of active sites on subsequent electrocatalysts. As shown in Figure S5 (Supporting Information), a‐Ni‐MeIM‐Co‐0.01 exhibited a much larger specific surface area and a new porous structure formed after reacting with Co^2+^.

Subsequently, one‐step vapor phase fluorination method was used to construct NiCo bimetallic fluoride. To our surprise, the as‐obtained Ni_0.42_Co_0.58_F_2_‐G presented a clear single characteristic peak of hexagonal phase metal fluoride with no other spurious peaks other than those from nickel fluoride and cobalt fluoride, indicating the successful synthesis of NiCo bimetallic fluoride (Figure 1e). In addition, its nitrogen adsorption and desorption isotherms manifested a distinct type IV isotherm (H3), indicating the presence of abundant mesoporous structures (Figure 1f,g).^[^ ^20^ ^]^

**Figure 2:**
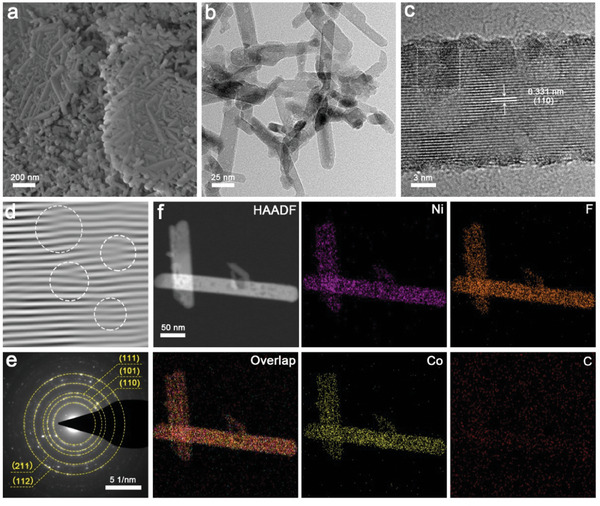
Morphological and structure characterization of Ni_0.42_Co_0.58_F_2_‐G. a) SEM, b) TEM, and c) HR‐TEM images of Ni_0.42_Co_0.58_F_2_‐G; d) atomic lattice image along the (110) crystal plane of the white dashed region in Figure 2c; e) selected area electron diffraction (SAED) and f) EDS spectra of Ni_0.42_Co_0.58_F_2_‐G.

The SEM and TEM were implemented to further explore the morphology and constructure of Ni_0.42_Co_0.58_F_2_‐G. As shown in **Figure** **2**a,b, Ni_0.42_Co_0.58_F_2_‐G appeared with a dense nanorods structure similar to NiF_2_, which differed from the agglomerated nanoparticle structure of CoF_2_, indicating that the presence of Ni was beneficial to the morphology stability (Figure S6, Supporting Information). Explicit lattice stripes can be observed throughout the rod structure from the HR‐TEM graph in Figure 2c, whose crystal plane spacing is 0.331 nm, slightly smaller than the (110) crystal plane of CoF_2_, which is also in line with the XRD patterns. Figure 2d representing the atomic lattice image along the (110) crystal plane and showed obvious lattice distortion, demonstrating the presence of abundant dislocations and stacking faults in Ni_0.42_Co_0.58_F_2_‐G.^[^ ^21^ ^]^ The above results indicated that Ni was successfully introduced into the lattice of CoF_2_. The SAED graph showed distinct diffraction rings corresponding to (110) (101) (111) (211) and (112) crystal planes of Ni_0.42_Co_0.58_F_2_‐G, respectively (Figure 2e). At the same time, the EDS elemental mapping established that Ni, Co, F, and C were uniformly distributed in the system (Figure 2f). In conclusion, NiCo bimetallic‐based fluoride Ni_0.42_Co_0.58_F_2_‐G was successfully prepared by solid–liquid exchange method as well as vapor phase fluorination method.

### 2.2 Electrochemical Test of Ni_0.42_Co_0.58_F_2_‐G and Comparison Samples

**Figure 3:**
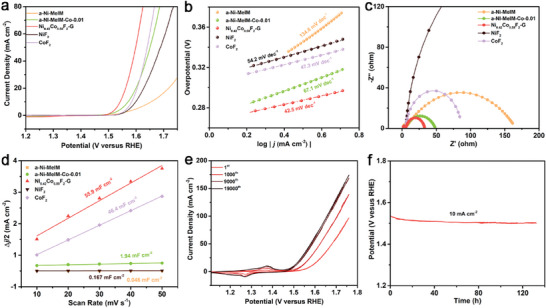
Electrochemistry test of different samples in 1 m KOH medium. a) Polarization curves, b) Steady‐state Tafel slopes, and c) Nyquist plots at 1.52 V versus RHE of Ni_0.42_C_o0.58_F_2_‐G and other electrocatalysts; d) The Δ*j*/2 at 1.22 V (vs RHE) as a function of the scan rate; e) Cyclic voltametric curves of Ni_0.42_C_o0.58_F_2_‐G after different cycles of CV; f) Chronopotentiometry test of Ni_0.42_C_o0.58_F_2_‐G at 10 mA cm^−2^.

The electrochemical performance of a‐Ni‐MeIM, a‐Ni‐MeIM‐Co‐0.01, Ni_0.42_Co_0.58_F_2_‐G, NiF_2_, and CoF_2_ were tested in 1 m KOH using the classical three‐electrode system. As shown in **Figure** **3**a, Ni_0.42_Co_0.58_F_2_‐G (313 mV@10 mA cm^−2^) exhibited outstanding OER performance, surpassed its complex precursors (427 mV of a‐Ni‐MeIM and 342 mV of a‐Ni‐MeIM‐Co‐0.01) and all the other fluorides samples (370 mV of NiF_2_ and 355 mV of CoF_2_). Due to the excellent performance of Ni_0.42_Co_0.58_F_2_‐G compared to the monometallic fluorides NiF_2_ and CoF_2_, it can be inferred that the contribution of bimetallic active center was constructed in Ni_0.42_Co_0.58_F_2_‐G. It can also be seen from the Tafel plot that Ni_0.42_Co_0.58_F_2_‐G showed lowest Tafel slope of 42.5 mV dec^−1^, demonstrating excellent electrochemical kinetics (Figure 3b). From the Nyquist plots in Figure 3c, it is clear that Ni_0.42_Co_0.58_F_2_‐G has the smallest contact resistance (*R*_ct_), indicating the fastest charge transfer kinetics and improved mass transfer at the surface of catalyst.^[^ ^22^ ^]^ The *C*_dl_ of the samples were measured to reveal the electrochemically active surface area (ECSA), and it can be seen that the *C*_dl_ of Ni_0.42_Co_0.58_F_2_‐G was much higher than the other catalysts, even 848 times more than a‐Ni‐MeIM, which demonstrated that large amounts of active sites were involved in electrochemical process on the surface of Ni_0.42_Co_0.58_F_2_‐G during the OER process (Figure 3d). An attractive phenomenon can be seen from the CV stability test, that is, the electrochemical activity of Ni_0.42_Co_0.58_F_2_‐G gradually rise with the increased CV cycles (CVs) and become stable after 9000 CVs, which showed that some changes probably occurred on the surface of electrocatalyst during electrochemical procedure (Figure 3e). The exploration about this will be discussed in subsequent section. Besides, Ni_0.42_Co_0.58_F_2_‐G remained stable for 10 000 CVs after arriving at its stable state, indicating its outstanding stability. Surprisingly, no obvious change of voltage was observed even after a long time of electrochemical oxygen evolution (Figure 3f). Moreover, in different current densities, Ni_0.42_Co_0.58_F_2_‐G exhibited dynamic stability with flexible response to different electrochemical conditions (Figure S9, Supporting Information). Together, all the conclusions above illustrate the outstanding electrochemical activity of Ni_0.42_Co_0.58_F_2_‐G, which is also better than most NiCo‐based electrocatalysts reported in recent years (Table S3, Supporting Information).

In the exploration of the impact of amorphous and crystalline complex precursors on their electrochemical performance, c‐Ni‐MeIM‐Co‐F‐G was synthesized by the similar method as Ni_0.42_Co_0.58_F_2_‐G, with the difference that a‐Ni‐MeIM‐Co‐α was replaced by c‐Ni‐MeIM‐Co‐0.01. It is found that c‐Ni‐MeIM‐Co‐0.01 still maintained strong crystallinity, indicating the difficulty of ionic exchange between Co^2+^ and c‐Ni‐MeIM, which is completely different from that of the amorphous a‐Ni‐MeIM‐Co‐0.01. After treatment with gas phase fluorination method, the peak of c‐Ni‐MeIM‐Co‐0.01 is still very obvious and new peaks of NiF_2_ appeared, such incomplete fluorination results further demonstrated its difficulty in modification (Figure S10, Supporting Information). This is also simultaneously confirmed by the stable morphology of c‐Ni‐MeIM‐Co‐F‐G during total synthesis processes (Figure S11, Supporting Information). By comparing the electrochemical activity of Ni_0.42_Co_0.58_F_2_‐G and c‐Ni‐MeIM‐Co‐F‐G, it confirms the advantages of fluorides derived from short‐range ordered complexes once again (Figure S12, Supporting Information). Moreover, Ni_x_Co_y_F_2_‐L was synthesized by a traditional liquid phase method. It can be seen that Ni_x_Co_y_F_2_‐L exhibits agglomerated nanoparticle morphology with lower electrochemical activity, as shown in Figures S13 and S14 (Supporting Information). From XRD pattern, no obvious fluoride peak can be found, indicating that liquid phase fluorination is more difficult than gas phase fluorination method, proving that the vapor phase fluorination method exhibits significant advantage compared to traditional liquid phase synthesis methods (Figure S15, Supporting Information).^[^ ^23^ ^]^ The difficulty of aqueous fluorination is originated from the higher ligand field strength of H_2_O or OH^−^ than that of F^−^.

### 2.3 Insights into Surface Reconstruction During OER Based on XPS and High‐Resolution TEM

**Figure 4:**
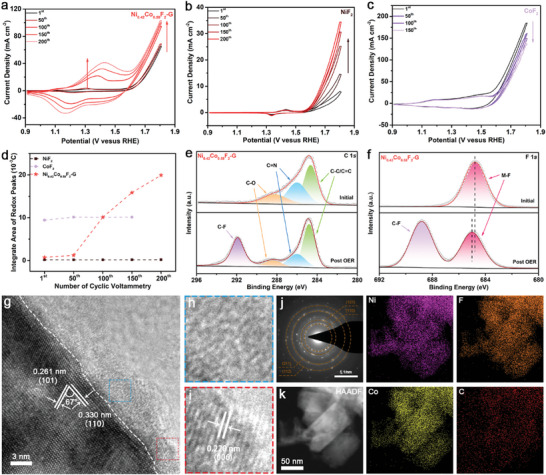
Cyclic voltametric curves of a) Ni_0.42_C_o0.58_F_2_‐G, b) NiF_2_ and c) CoF_2_ after different cycles of CV; d) Variation of redox peak area with CVs corresponding to Figure 4a–c; XPS high‐resolution spectra of e) C 1*s* and f) F 1*s* of Ni_0.42_C_o0.58_F_2_‐G before and after OER; g) HR‐TEM image of Ni_0.42_C_o0.58_F_2_‐G after electrochemistry stability test; h,i) correspond to the images in the blue dashed box and the red dashed box in Figure 4g, respectively; j) SAED image and k) corresponding EDS elemental mapping images of Ni_0.42_C_o0.58_F_2_‐G after electrochemistry stability test.

From the analysis of the electrochemical section, it can be tentatively deduced that surface reconstruction developed as the electrocatalytic process proceeds. As displayed in **Figure** **4**a, there is significant uplift (expansion) on the CV curves of Ni_0.42_Co_0.58_F_2_‐G, exhibiting not only more distinct redox peaks as well as gradual increment of peak area, but also increasing current density of OER. In comparison, although redox peaks can also be observed in CoF_2_ and NiF_2_, there is no significant change in their peak areas (Figure 4b,c). It is noted that the OER current density of Ni_0.42_Co_0.58_F_2_‐G displayed an increasing trend with the increment of CVs, which is similar to that ofNiF_2_, illustrating that the presence of Ni favors the stabilization of the products after surface reconstruction, highlighting the advantages of bimetallic center over monometallic ones. In recent years, Zhou et al. reported that the redox peak area is proportional to the number of active sites.^[^ ^15b^ ^]^ As shown in Figure 4d, with the electrochemical activation, the number of active sites of Ni_0.42_Co_0.58_F_2_‐G increased significantly, demonstrating that in addition to the electrochemical oxidation of Ni^2+^ and Co^2+^ under the high potential conditions during OER, some other changes also increased the number of active sites. Figure 4e represented the XPS high‐resolution C 1*s* spectra of Ni_0.42_Co_0.58_F_2_‐G before and after OER, which exhibited three main fitted peaks at 284.6, 286.0, and 288.2 eV, corresponding to C─C/C═C, C═N and C─O groups respectively.^[^ ^24^ ^]^ Surprisingly, a clear characteristic peak at 291.9 eV appeared, which can be determined to be C─F. Apparently, it is the highest electronegativity with strong induction effect of F, resulting in such a significant positive displacement of the binding energy of C 1*s*. Also, the peak corresponding to C─F bond was observed in the F 1s spectrum after OER, located at 688.8 eV, distinguished from M─F. A larger binding energy of C─F than M─F is also attributed to the higher electronegativity of C compared to the metal (Figure 4f). The above results demonstrated that a migration of F from metal surface to C occurred during OER, which undoubtedly exposed more metal sites accessible to the electrolyte. It is noteworthy that the intensity of characteristic peak corresponding to M─F underwent a significant decrease after OER, whose binding energy was positively shifted to some extent, resulting from the high‐potential oxidation by OER and the breakage of the M─F bond due to F migration. These results further proved that the surface reconstruction during OER caused the migration of F and enlarged the ECSA, which was also confirmed by the previous result of outstanding *C*_dl_ and increasing number of active sites. The XRD pattern showed that Ni_0.42_Co_0.58_F_2_‐G still maintained a well‐crystalline phase structure with a slight decrease of intensity after OER long time stability test, which also suggested that the catalyst was only reconstructed on the surface and no other apparent transformation occurred inside (Figure S16, Supporting Information).

The structure of post OER catalyst was further analyzed using HRTEM (Figure 4g). An obvious heterostructure consisting of crystalline and amorphous regions can be seen in the figure, with the crystalline region corresponding to NiCo bimetallic fluoride and the amorphous region exhibiting faint lattice stripes corresponding to the (006) crystal plane of CoOOH. The SAED graph displayed distinct diffraction rings correspond to the (110) (101) (211) and (112) crystal facets of Ni_0.42_Co_0.58_F_2_‐G, respectively (Figure 4j), which also agrees with the XRD result. Meanwhile, the elemental mapping graphs showed that Ni, Co, F, and C were evenly distributed in catalyst (Figure 4k). The high‐resolution Ni 2*p* and Co 2*p* XPS spectra of Ni_0.42_Co_0.58_F_2_‐G before and after OER were shown in Figure S17 (Supporting Information). No obvious shift of peaks position of both Ni 2*p* and Co 2*p* was observed after OER. This could be from two reasons. First, the reconstruction occurred only on the very thin surface of Ni_0.42_Co_0.58_F_2_‐G, which causes very slight change on valance state. Second, due to the large electronegativity of fluorine, the binding energies of Ni 2*p* and Co 2*p* peaks in fluorides located on higher position, which were similar to the NiCo‐based (oxy)hydroxide. In summary, Ni_0.42_Co_0.58_F_2_‐G maintained the stability of valance states during OER process, the OER process accelerated the surface reconstruction. These results above revealed that the catalyst suffered a certain degree of surface reconstruction during the OER process and formed Ni‐substituted CoOOH with amorphous state on the surface, which is not only the real active site during the OER, but also ensures the stable crystallinity inside the catalyst.

### 2.4 Valence State and Electronic Structure Analysis in Bimetallic Fluorides

**Figure 5:**
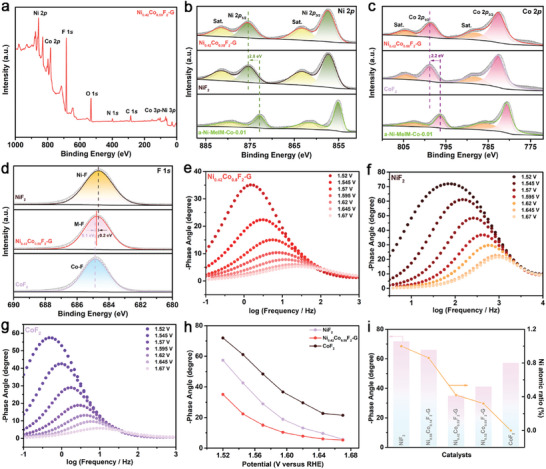
a) XPS survey spectrum of Ni_0.42_Co_0.58_F_2_‐G; XPS high‐resolution spectra of b) Ni 2*p*, c) Co 2*p* and d) F 1*s* of NiF_2_, CoF_2,_ and Ni_0.42_Co_0.58_F_2_‐G; Bode plots of e) Ni_0.42_Co_0.58_F_2_‐G,f) NiF_2_ and g) CoF_2_ at potential range from 1.52 to 1.67 V (vs RHE); h) Potential dependence of the phase angle of NiF_2_, CoF_2,_ and Ni_0.42_Co_0.58_F_2_‐G; i) phase angle at the potential of 1.52 V of fluorides with different NiCo atomic ratios.

The reasons for the more efficient surface reconstruction of NiCo bimetallic fluoride compared to monometallic fluorides were further analyzed from the valence and electronic perspectives. The XPS survey spectrum shows the presence of Ni, Co, F, and C in N_i0.42_Co_0.58_F_2_‐G, which is also consistent with the EDS results (**Figure** **5**a). It can be seen from Ni 2*p* spectrum in Figure 5b that the Ni 2*p* peak of N_i0.42_Co_0.58_F_2_‐G was similar to that of NiF_2_ and had a very significant positive shift compared to that of a‐Ni‐MeIM‐Co‐0.01. The same conclusion can be drawn from the Co 2*p* spectrum (Figure 5c). This is due to the fact that fluorine has the highest electronegativity and such strong induction effect greatly reduces the electron cloud density of Ni and Co, thus raising the valence state of metal ions, which can also accelerate the surface reconstruction of the catalyst.^[^ ^25^ ^]^ F 1*s* spectrum were used to observe the changes of Ni and Co valence states in Ni_0.42_Co_0.58_F_2_‐G. As shown in the Figure 5d, the position of the peak representing M─F in N_i0.42_Co_0.58_F_2_‐G was between Ni─F in NiF_2_ and Co─F in CoF_2_ with a gap of 0.1–0.2 eV, demonstrating that the presence of NiCo bimetallic center, which can modulate the electronic structure that may contribute to surface reconstruction. Besides, Figure 5e–g exhibited the Bode plots of monometallic fluoride and bimetallic fluoride. The significant response signals of N_i0.42_Co_0.58_F_2_‐G and CoF_2_ in the low frequency region are caused by the heterogeneous charge distribution of the surface oxide species, corresponding to the process of OER. Specifically, the response signal of NiF_2_ was biased toward the high‐frequency region, corresponding to the oxidation from Ni^2+^ to Ni^3+^, which not only explains the higher OER activity of the former compared to the latter, but is also consistent with that reported in the literature.^[^ ^26^ ^]^ It is obvious in the Figure 5h that N_i0.42_Co_0.58_F_2_‐G has the smallest phase angle at all voltages, which means that more electrons are involved in the OER reaction.^[^ ^27^ ^]^ Figure 5i illustrated a near volcano‐type relationship between the phase angle and the NiCo ratio in bimetallic fluoride, which meant that Ni_0.42_Co_0.58_F_2_‐G has the best NiCo ratio with optimized electronic structure. The above analysis of the electronic structure and valence states not only revealed the important role of the appropriate ratio of NiCo bimetallic center for the regulation of the electronic structure, but also further explained the increase of electrochemical active site due to the F migration during OER.

### 2.5 Density Function Theory Calculations

**Figure 6:**
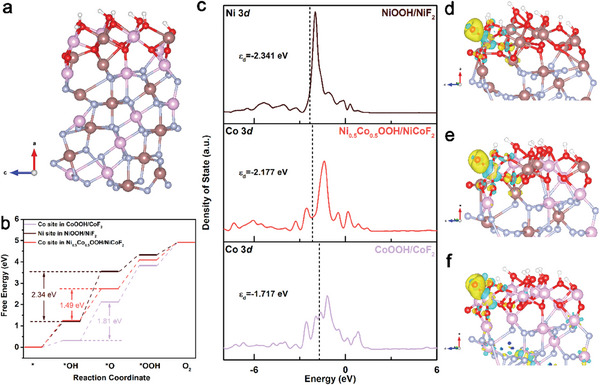
a) The crystal structure of Ni_0.5_Co_0.5_OOH/NiCoF_2_; b) The calculated Gibbs free energy diagram for the four steps of OER over NiOOH/NiF_2_, Ni_0.5_Co_0.5_OOH/NiCoF_2_ and CoOOH/CoF_2_; c) DOS of NiOOH/NiF_2_ regarding the Ni 3d orbital as well as CoOOH/CoF_2_ and Ni_0.5_Co_0.5_OOH/NiCoF_2_ regarding the Co 3d orbitals; Charge density difference for ^*^O intermediate on d) NiOOH/NiF_2_, e) Ni_0.5_Co_0.5_OOH/NiCoF_2_ and f) CoOOH/CoF_2_ (the blue and yellow show the electron losing and gaining, respectively).

In order to obtain the profound insight into the surface reconstruction phenomenon during OER, density function theory (DFT) calculations were carried out. As shown in **Figure** **6**a and Figure S18 (Supporting Information), three heterojunction models were constructed and denoted as NiOOH/NiF_2_, CoOOH/CoF_2_, and Ni_0.5_Co_0.5_OOH/NiCoF_2_, corresponding to NiF_2_, CoF_2_, and Ni_0.42_Co_0.58_F_2_‐G after surface reconstruction, respectively. Figure 6b reveals the variation of Gibbs free energy for different catalysts in the elementary OER steps. It can be seen that the formation of ^*^O is the rate determining step (RDS) of OER. Significantly, the reaction energy barrier of Ni_0.5_Co_0.5_OOH/NiCoF_2_ is 1.49 eV, which is lower than that of NiOOH/NiF_2_ (2.34 eV) and CoOOH/CoF_2_ (1.81 eV). The results above also agree well with the experimentally obtained overpotential trends, indicating the reliability of the theoretical calculations. In order to explore the effect of heterostructure resulted from surface reconstruction during OER, DFT models of Ni_0.5_Co_0.5_OOH and NiCoF_2_ were established (Figures S22 and S23, Supporting Information). As shown in Figure S24 (Supporting Information), NiCoF_2_ displayed highest reaction energy barrier of 5.27 eV. This is due to the fact that the adsorption of oxygen‐containing intermediates on NiCoF_2_ is too strong, making the desorption of oxygen difficult. Moreover, the reaction energy barrier of Ni_0.5_Co_0.5_OOH (2.88 eV) is also higher than that of Ni_0.5_Co_0.5_OOH/NiCoF_2_, whose RDS is the generation of ^*^OOH. The results above suggest that the building of heterostructure can effectively modulate the RDS of OER and optimize the energy barrier, which also illustrates the critical role of surface reconstruction for the pre‐catalysts. Subsequently, the density of state (DOS) of Ni 3*d* orbital in NiOOH/NiF_2_ and Co 3*d* orbital in CoOOH/CoF_2_ and in Ni_0.5_Co_0.5_OOH/NiCoF_2_ were analyzed, respectively. All of them exhibit distinct metallic characteristics of Ni or Co. It is well known that the overlapping of 3*d* orbital of metal to the 2*p* orbital of O produces the bonding orbital and antibonding orbital, and a higher energy of *d* band center means that the lower population of electrons filled in the antibonding orbital, the stronger the adsorption (interaction) of metal to the oxygen‐containing intermediate. Figure 6c illustrates that Co site in CoOOH/CoF_2_ has the highest *d* band center (−1.717 eV), leading to a tenacious adsorption between the metal sites and oxygenated species. It is noteworthy that such strong adsorption is detrimental to the subsequent evolutionary reactions, leading to a higher RDS energy barrier. To our surprise, the Co site in Ni_0.5_Co_0.5_OOH/NiCoF_2_ exhibited balanced *d* band center (−2.177 eV), facilitating both of the adsorption and evolution of oxygen‐containing intermediates, which is also consistent with the results of Gibbs free energy diagram. Furthermore, charge density differences (CDD) of samples were determined to explored the electron distribution during OER (Figure 6d–f). Notably, the Co site in Ni_0.5_Co_0.5_OOH/NiCoF_2_ has more electron deficiencies compared to the other two metal sites, further suggesting that the construction of bimetallic center can induce charge redistribution and generate higher valence metal sites. The latter not only are the actual active site, but also are beneficial to accelerating surface reconstruction and promoting OER kinetics.

## 3 Conclusion

In summary, NiCo‐based nanoarray a‐Ni‐MeIM‐Co‐0.01 precursors were first designed and synthesized by a simple solid–liquid exchange method with a considerable specific surface area, which facilitated the subsequent construction of NiCo bimetallic fluoride N_i0.42_Co_0.58_F_2_‐G with a high electrochemical active surface area. Meanwhile, the fluoride produced by the vapor phase fluorination method has higher crystallinity compared with the conventional liquid‐phase synthesis method. The highly efficient surface reconstruction accelerated by the bimetallic center regulated electronic structure during the electrochemical process, and the high electrochemical activity area due to the fluorine migration contributed not only to the excellent electrochemical performance of Ni_0.42_Co_0.58_F_2_‐G, but also secured its electrochemical stability. DFT calculations show that the redistribution of electrons due to surface reconfiguration during electrochemical processes reduces the energy barrier of rate determining step and facilitates OER. The present work is also helpful for the future design and preparation of effective electrochemical surface reconstruction electrocatalysts.

## 4 Experimental Section

### Materials

Nickel nitrate hexahydrate (Ni(NO_3_)_2_·6H_2_O, AR,), cobalt nitrate hexahydrate (Co(NO_3_)_2_·6H_2_O, AR,), Cetyltrimethylammonium bromide (CTAB, AR), ethanol (C_2_H_5_OH, AR) and ammonium fluoride (NH_4_F, AR) were obtained from Sinopharm Chemical Reagent Co. Ltd. 2‐Methylimidazole (2‐MeIM) was purchased from Aladdin. In particular, the water used for all experiments was purified ultra‐pure water (up water), with electrical resistivity of 18.25 MΩ cm^−1^. All other chemicals were utilized directly in experiments.

### Synthesis of a‐Ni‐MeIM

At room temperature, 292 mg of (Ni(NO_3_)_2_·6H_2_O and 4 mg of CTAB were dissolved in 10 mL of up H_2_O to form homogenous solution as solution A. Then, 4.54 g of 2‐MeIM was solubilized in 70 mL of up H_2_O and stirred continuously as solution B. The above solutions were quickly mixed and stirring vigorously for 1 h, left to stratify, the supernatant was discarded and the remaining solution was centrifuged. The precipitate was cleaned three times with ethanol and dried in a desiccator for 12 h. The dried solid was ground using a mortar to obtain a yellow powder and marked as a‐Ni‐MeIM. The corresponding c‐Ni‐MeIM was synthesized according to the method reported

### Synthesis of a‐Ni‐MeIM‐Co‐α

A facile solid–liquid contact modification method was unitized for the preparation of a/c‐Ni‐MeIM‐Co‐α. First of all, different amounts of Co(NO_3_)_2_‐6H_2_O and 2 mg CTAB were dissolved in 30 mL up H_2_O to prepare a series of Co^2+^ solutions with a concentration gradient. After that, 50 mg of a/c‐Ni‐MeIM were added to Co^2+^ solution and stirred continuously for 0.5 h, and supernatant was separated for the first time after centrifugation, and the precipitate was cleaned few times by ethanol and dried in a desiccator for 12 h. Depending on the concentration of Co^2+^, products were recorded as a‐Ni‐MeIM‐Co‐H_2_O, a‐Ni‐MeIM‐Co‐0.002, a‐Ni‐MeIM‐Co‐0.01, a‐Ni‐MeIM‐Co‐0.04.

### Synthesis of NixCoyF2‐G

Ni_x_Co_y_F_2_‐G were synthesized using a one‐step vapor phase fluorination method. 20 mg of a‐Ni‐MeIM‐Co‐α was placed downstream of the porcelain boat as well as 800 mg of NH_4_F was laid upstream of the porcelain boat, the whole reaction system was set in a tube furnace filling with N_2_ and reacted at 400 °C for 1 h (heating rate 10 °C min^−1^). Based on the ICP‐AES results, the samples were recorded as Ni_0.86_Co_0.14_F_2_‐G, Ni_0.42_Co_0.58_F_2_‐G, and Ni_0.32_Co_0.68_F_2_‐G, respectively, according to their NiCo ratios (Table S1, Supporting Information).

### Synthesis of Control Samples

As a control, Ni_x_Co_y_F_2_‐L and c‐Ni‐MeIM was prepared refer to the approach reported in the literature.^[^ ^19^, ^23^ ^]^ The synthesis of c‐Ni‐MeIM‐Co‐0.01 was similar to that of a‐Ni‐MeIM‐Co‐0.01, except that a‐Ni‐MeIM was replaced by c‐Ni‐MeIM. NiF_2_, CoF_2,_ and c‐Ni‐MeIM‐Co‐F‐G were synthesized in basically the same way as Ni_x_Co_y_F_2_‐G, with the difference that a‐Ni‐MeIM‐Co‐α was replaced by a‐Ni‐MeIM, ZIF‐67 and c‐Ni‐MeIM‐Co‐0.01, respectively.

### Structural Characterization

Scanning electron microscopy (SEM) graphs were taken by Zeiss SIGMA. Transmission electron microscopy (TEM) coupled with EDS, SAED, and high‐resolution TEM were conducted by FEI Tecnai G2 F30 S‐TWIN. X‐ray diffraction patterns were obtained by Bruker D8 Advance and Rigaku Miniflex600. N_2_ adsorption–desorption analysis was undertaken by TriStar II 3020. Quantitative elemental analyses were measured by inductively coupled plasma‐atomic emission spectroscopy (Agilent 5110) and Atomic Absorption Spectroscopy (contrAA700). Fourier transform infrared spectroscopy was conducted by Thermo FTIR5700. X‐ray photoelectron spectroscopy (XPS) analyses were carried out by Thermo Fisher Scientific ESCALAB250Xi.

### Electrochemical Measurement

All the tests were achieved on a CHI‐760e work station. The rotational speed of rotating disk electrode was 1600 rpm s^−1^. The working electrode, reference electrode, and counter electrode were glassy carbon electrode, HgO/Hg, and Pt sheet, respectively. 5 mg samples and 0.02 mL 5 wt.% Nafion solution were dissolved in 0.98 mL absolute isopropanol via sonication to produce a homogeneous catalysts ink. Then, 12 µL of the catalyst ink was dropped onto the glassy carbon electrode and dried in the air. The electrochemical activation process consists of cyclic voltammetry (CV) scans in the voltage range of 0.92–1.82 V (vs RHE) with a sweep rate of 500 mV s^−1^ for 50 cycles was conduct to activate and clear up the surface. Afterwards, linear scanning voltammetry (LSV) curves with 80% iR correction were applied to test the electrochemical performance of the catalysts, whose scan rate was 5 mV s^−1^. The Tafel slope depends on the logarithmic relationship between current density and voltage. Electrochemical impedance spectroscopy (EIS) was measured in the frequency range of 0.01–100 kHz from 1.52 to 1.67 V versus RHE. The electrochemical double layer capacitance (*C*_dl_) was conducted by cyclic voltammetry curves in the non‐Faraday region (1.17–1.27 V vs RHE). A certain amount of catalyst loaded on nickel foam was utilized as working electrode directly for electrochemical stability test. The test voltage (*E*_HgO/Hg_) was converted to a voltage compared to the reversible hydrogen electrode (vs RHE) by the following equation: *E_RHE_* = *E*_*HgO*/*Hg*_  + 0.059 × *pH* + 0.098.

## Conflict of Interest

The authors declare no conflict of interest.

## Supporting information

Supporting Information

## Data Availability

The data that support the findings of this study are available in the supplementary material of this article.

## References

[1] a) J. Zhang , Y. Ye , Z. Wang , Y. Xu , L. Gui , B. He , L. Zhao , Adv. Sci. 2022, 9, 2201916; 10.1002/advs.202201916 PMC9507342 35869034

[2] a) H. Wang , T. Zhai , Y. Wu , T. Zhou , B. Zhou , C. Shang , Z. Guo , Adv. Sci. 2023, 10, 2301706; 10.1002/advs.202301706 PMC10401147 37253121

[3] H. Chen , X. Liang , Y. Liu , X. Ai , T. Asefa , X. Zou , Adv. Mater. 2020, 32, 2002435. 10.1002/adma.202002435 32666550

[4] a) I. C. Man , H.‐Y. Su , F. Calle‐Vallejo , H. A. Hansen , J. I. Martínez , N. G. Inoglu , J. Kitchin , T. F. Jaramillo , J. K. Nørskov , J. Rossmeisl , ChemCatChem. 2011, 3, 1159;

[5] a) X. Zhang , Z. Luo , Z. Zhou , Y. Wang , Z. Cui , Z. Gao , J. Shi , T. Cao , X. Fan , J. Electroanal. Chem. 2022, 922, 116731;

[6] a) C. Das , N. Sinha , P. Roy , Small 2022, 18, 2202033; 10.1002/smll.202202033 35703063

[7] X. Ma , K. Li , X. Zhang , B. Wei , H. Yang , L. Liu , M. Zhang , X. Zhang , Y. Chen , J. Mater. Chem. A 2019, 7, 14904.

[8] a) K. Kawashima , R. A. Márquez‐Montes , H. Li , K. Shin , C. L. Cao , K. M. Vo , Y. J. Son , B. R. Wygant , A. Chunangad , D. H. Youn , G. Henkelman , V. H. Ramos‐Sánchez , C. B. Mullins , Mater. Adv. 2021, 2, 2299;

[9] a) J. Yang , J. K. Cooper , F. M. Toma , K. A. Walczak , M. Favaro , J. W. Beeman , L. H. Hess , C. Wang , C. Zhu , S. Gul , J. Yano , C. Kisielowski , A. Schwartzberg , I. D. Sharp , Nat. Mater. 2017, 16, 335; 27820814 10.1038/nmat4794

[10] J. Liu , Y. Ji , J. Nai , X. Niu , Y. Luo , L. Guo , S. Yang , Energy Environ. Sci. 2018, 11, 1736.

[11] a) X. Wei , Y. Zhang , H. He , L. Peng , S. Xiao , S. Yao , P. Xiao , Chem. Commun. 2019, 55, 10896; 10.1039/c9cc05225a 31436763

[12] a) X. Yu , S. Xu , X. Liu , X. Cheng , Y. Du , Q. Wu , J. Alloy. Compd. 2021, 878, 160388;

[13] Z. Liu , H. Liu , X. Gu , L. Feng , Chem. Eng. J. 2020, 397, 125500.

[14] a) W. Li , A. Murisana , Q. Zhang , S. Wang , G. De , Electrochem. Commun. 2022, 141, 107363;

[15] a) H. Jia , N. Yao , J. Zhu , W. Luo , Chem. Eur. J. 2023, 29, 202203073; 10.1002/chem.202203073 36367365

[16] a) R. Paul , Q. Zhai , A. K. Roy , L. Dai , Interdiscip. Mater. 2022, 1, 28;

[17] a) Q. Wang , Z. Zhang , S. Shi , F. Wu , Z. Zhang , G. Li , Y. Suo , J. Electroanal. Chem. 2021, 894, 115397;

[18] F. Wang , Y.‐X. Tan , H. Yang , H.‐X. Zhang , Y. Kang , J. Zhang , Chem. Commun. 2011, 47, 5828. 10.1039/c1cc10829h 21487610

[19] A. H. A. Rahim , S. N. F. Yusuf , S. R. Majid , Z. Osman , J. Appl. Electrochem. 2021, 52, 159.

[20] a) M. Kruk , M. Jaroniec , Chem. Mater 2001, 13, 3169;

[21] a) K. Huang , D. Peng , Z. Yao , J. Xia , B. Zhang , H. Liu , Z. Chen , F. Wu , J. Wu , Y. Huang , Chem. Eng. J. 2021, 425, 131533;

[22] A. Saad , Y. Gao , K. A. Owusu , W. Liu , Y. Wu , A. Ramiere , H. Guo , P. Tsiakaras , X. Cai , Small 2022, 18, 2104303. 10.1002/smll.202104303 35142066

[23] P.‐Y. Lee , L.‐Y. Lin , Energy 2022, 239, 122129.

[24] a) Z. Chen , Y. Ha , H. Jia , X. Yan , M. Chen , M. Liu , R. Wu , Adv. Energy Mater. 2019, 9, 1803918;

[25] L. Li , X. Cao , J. Huo , J. Qu , W. Chen , C. Liu , Y. Zhao , H. Liu , G. Wang , J. Energy Chem. 2023, 76, 195.

[26] Y. Qi , Y. Zhang , L. Yang , Y. Zhao , Y. Zhu , H. Jiang , C. Li , Nat. Commun. 2022, 13, 4602. 35933480 10.1038/s41467-022-32443-5 PMC9357015

[27] Y. Wang , B. Liu , X. Shen , H. Arandiyan , T. Zhao , Y. Li , M. Garbrecht , Z. Su , L. Han , A. Tricoli , C. Zhao , Adv. Energy Mater. 2021, 11, 2003759.

