# Supplementary material for: Surface Reconstruction Facilitated by Fluorine Migration and Bimetallic Center in NiCo Bimetallic Fluoride Toward Oxygen Evolution Reaction
Source: Adv Sci (Weinh). 2023 Dec 3;11(6):2306758. doi: 10.1002/advs.202306758 (PMC10853698; doi:10.1002/advs.202306758)
Supplement: Supplementary file 1 — Supporting Information [file ADVS-11-2306758-s001.pdf]

ADVANCED SCIENCE
Open Access

Supporting Information

for *Adv. Sci.*, DOI 10.1002/advs.202306758

Surface Reconstruction Facilitated by Fluorine Migration and Bimetallic Center in NiCo Bimetallic Fluoride Toward Oxygen Evolution Reaction

Zhenhang Xu, Wei Zuo, Yueying Yu, Jinyan Liu, Gongzhen Cheng and Pingping Zhao**

Supporting Information for

Surface Reconstruction Facilitated by Fluorine Migration and Bimetallic Center in NiCo Bimetallic Fluoride Toward Oxygen Evolution Reaction

Zhenhang Xu, Wei Zuo, Yueying Yu, Jinyan Liu, Gongzhen Cheng,* and Pingping Zhao*

Computational Methods

DFT calculations were performed by using the Vienna Ab-initio Simulation Package (VASP). The exchange–correlation interactions were described by generalized gradient approximation (GGA) with the Perdew–Burke–Ernzerhof (PBE) functiona. Spin-polarization was included in all the calculations and a damped van der Waals correction was incorporated using Grimme's scheme to better describe the non-bonding interactions. A plane wave cut-off energy of 500 eV was used, and a 3×3×1 Monkhorst-Pack grid k-points was employed. The residual force and energy on each atom during structure relaxation were converged to 0.005 eV $Å^{-1}$ and 10^{-5} eV, respectively. To account for the effect of localized d electrons of Ni and Co ions, a Hubbard U correction was introduced with U values of 3.8 and 4.0 eV, respectively.

The OER pathway was described as the adsorption of successive intermediate species on the catalyst and the relevant reaction energies were as follows (*Eq.* 1 ~ *Eq.* 4):

1. $OH^- + M \rightarrow *OH\text{-}cat + e^-$
2. $*OH\text{-}M + OH^- \rightarrow *O\text{-}cat + H_2O + e^-$
3. $*O\text{-}M + OH^- \rightarrow *OOH\text{-}cat + e^-$
4. $*OOH\text{-}M + OH^- \rightarrow O_2\uparrow + H_2O + e^-$

The "M" represented the active site when OER occurred. The "*OH", "*O", "*OOH" represented the intermediate species adsorbed on the active sites. In order to evaluate OER activity, we calculated the free energy (ΔG_1 ~ΔG_4) using the computational standard hydrogen electrode model. The free energy calculation could be obtained as follows:

$\Delta G_1 = G_{OH\text{-}cat} - G_{cat} - G_{H2O} + 1/2G_{H2} - eU + K_BTLn10\cdot pH$

$\Delta G_2 = G_{O\text{-}cat} - G_{OH\text{-}cat} + 1/2G_{H2} - eU + K_BTLn10\cdot pH$

$\Delta G_3 = G_{OOH\text{-}cat} - G_{O\text{-}cat} - G_{H2O} + 1/2G_{H2} - eU + K_BTLn10\cdot pH$

$\Delta G_4 = 4.92 - \Delta G1 - \Delta G2 - \Delta G3$

It should be noted that -eU represented the free energy changes for one electron transfer where U was electrode potential respect to the standard hydrogen electrode. For pH ≠ 0, pH effected on free energy could be defined as $-K_BTLn10{\cdot}pH$, where K_B was Boltzman constant. ΔG_4 was calculated by 4.92 - ΔG1 - ΔG2 - ΔG3 to avoid calculating the O_2 adsorption and desorption. It was known that the DFT calculation might not accurately describe the free energy of O_2 molecule in the gas phase and hence we used H_2O and H_2 as reference and from there we extracted the free energy of O_2 through the reaction $O_2 + 4(H^+ + e^-) \rightarrow 2H_2O$. The equilibrium potential for this reaction was 1.23 V and since it was a four-electron transfer reaction, the full energy was 4 × 1.23 = 4.92 eV. This analysis was based on the scheme developed by Norskov's group. The overpotential of OER in this mechanism was defined as $\eta_{OER} = \max(\Delta G_{OER}/e) - 1.23$ V.

Supplementary Figures

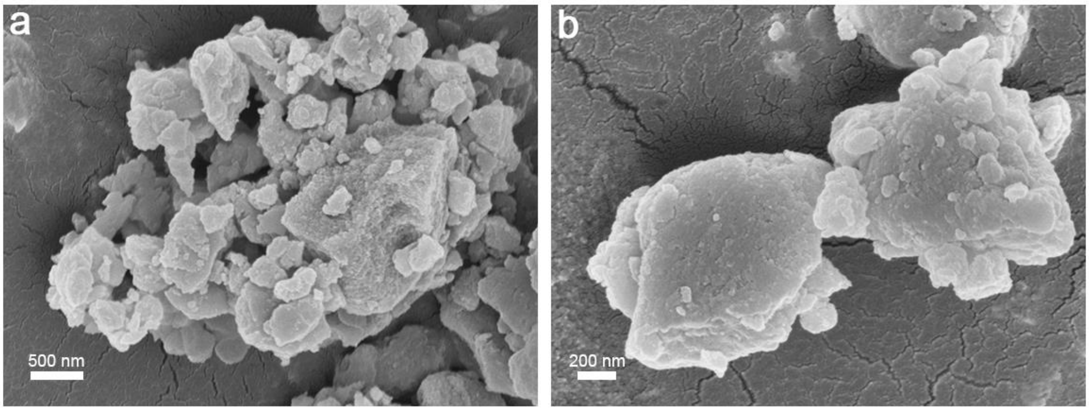

Figure S1. SEM images of a-Ni-MeIM.

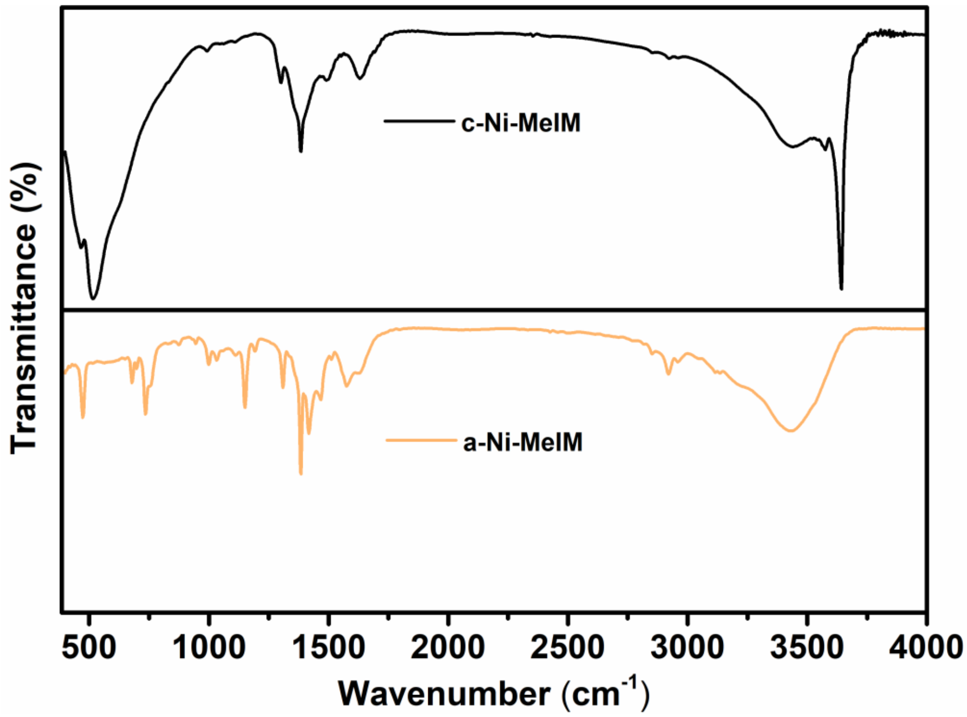

Figure S2. FTIR spectra of a-Ni-MeIM and c-Ni-MeIM.

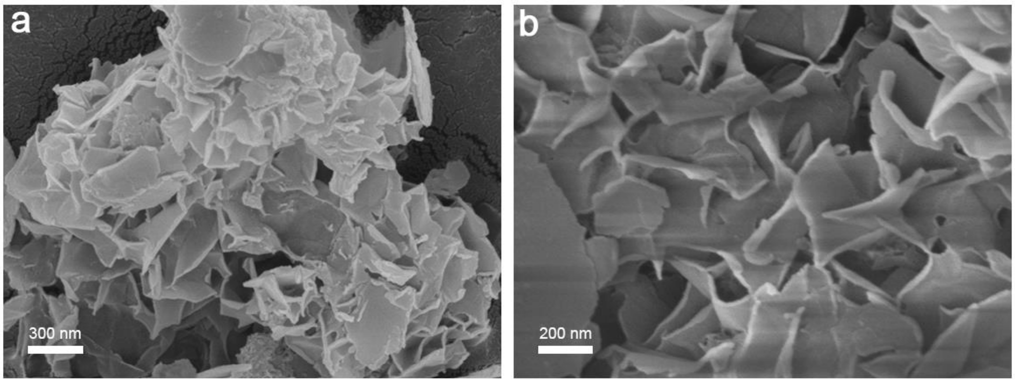

Figure S3. SEM images of a-Ni-MeIM-Co-0.01.

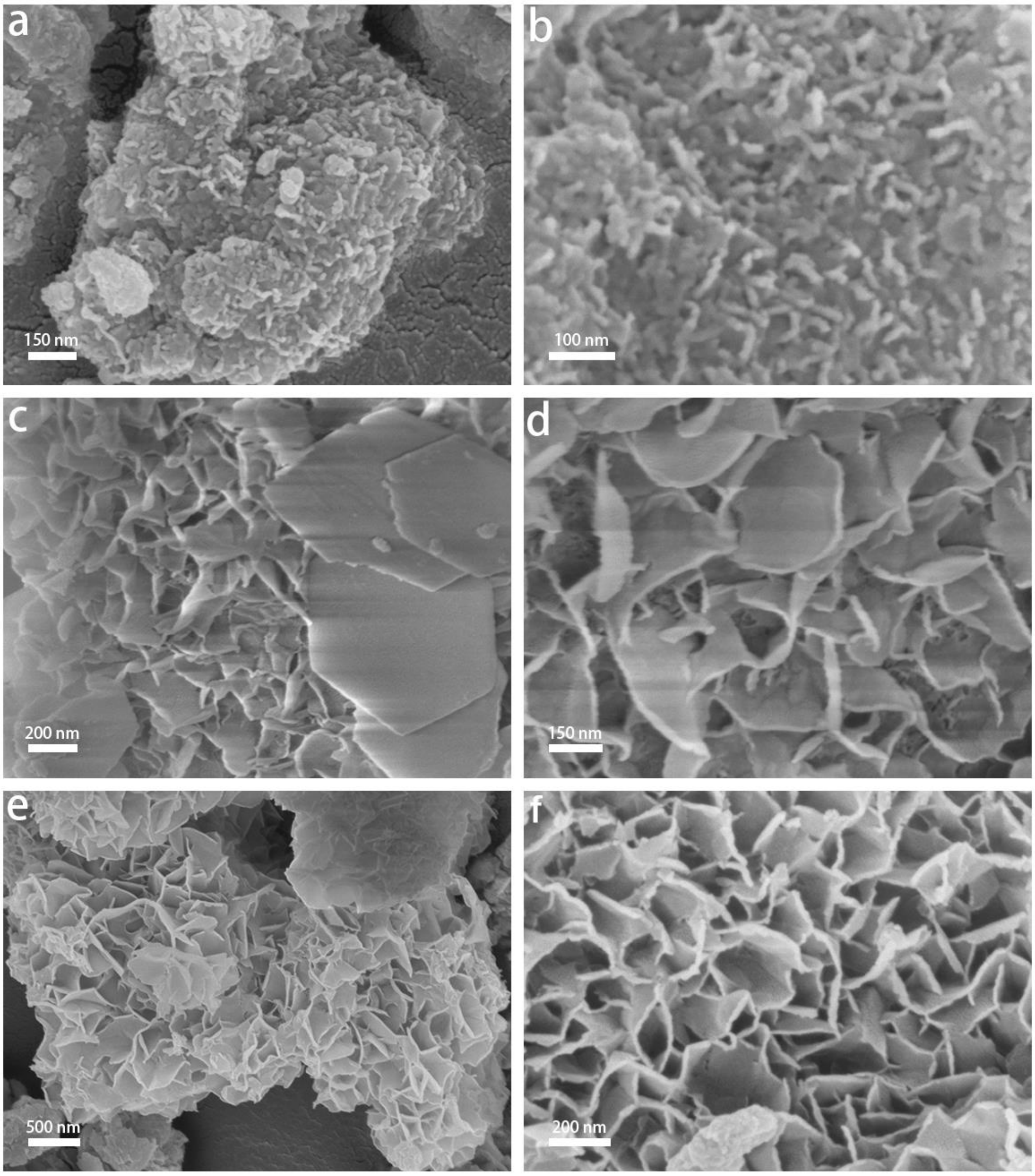

Figure S4. SEM images of a) and b) a-Ni-MeIM-Co-H_2O, c) and d) a-Ni-MeIM-Co-0.002, e) and f) a-Ni-MeIM-Co-0.04.

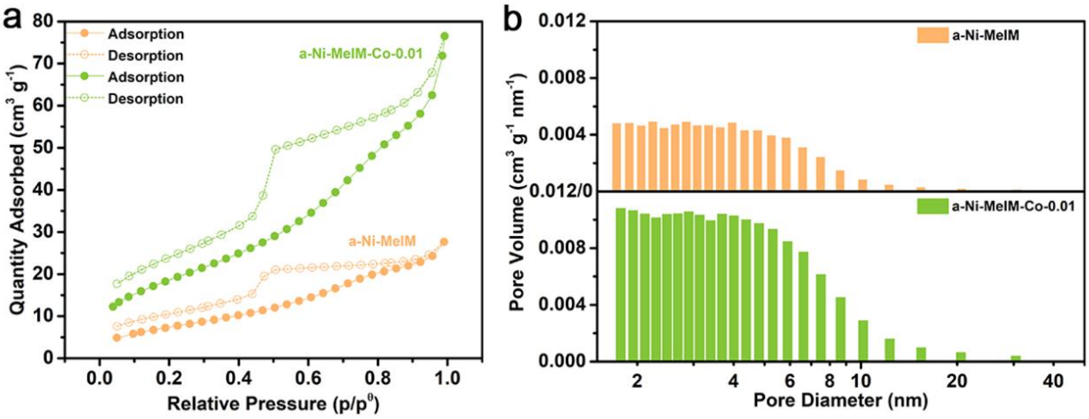

Figure S5. a) N_2 adsorption–desorption isotherms and b) pore size distribution curves of a-Ni-MeIM and a-Ni-MeIM-Co-0.01.

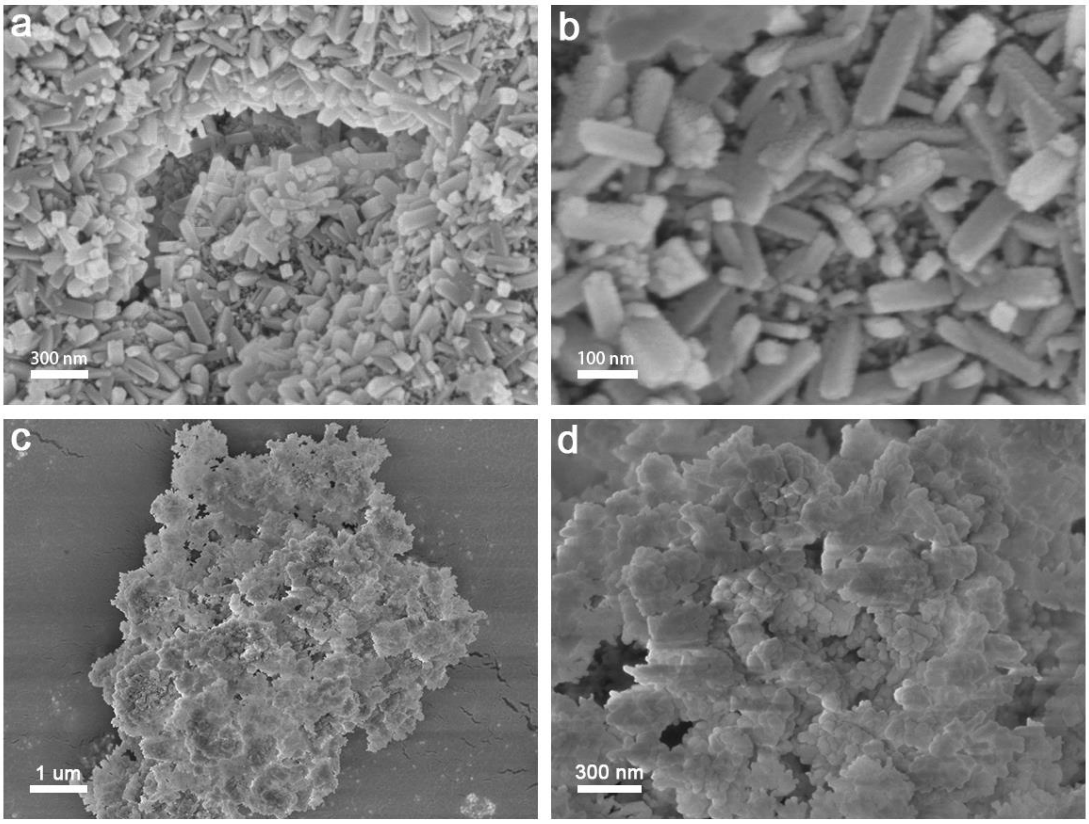

Figure S6. SEM images of a) and b) NiF_2, c) and d) CoF_2.

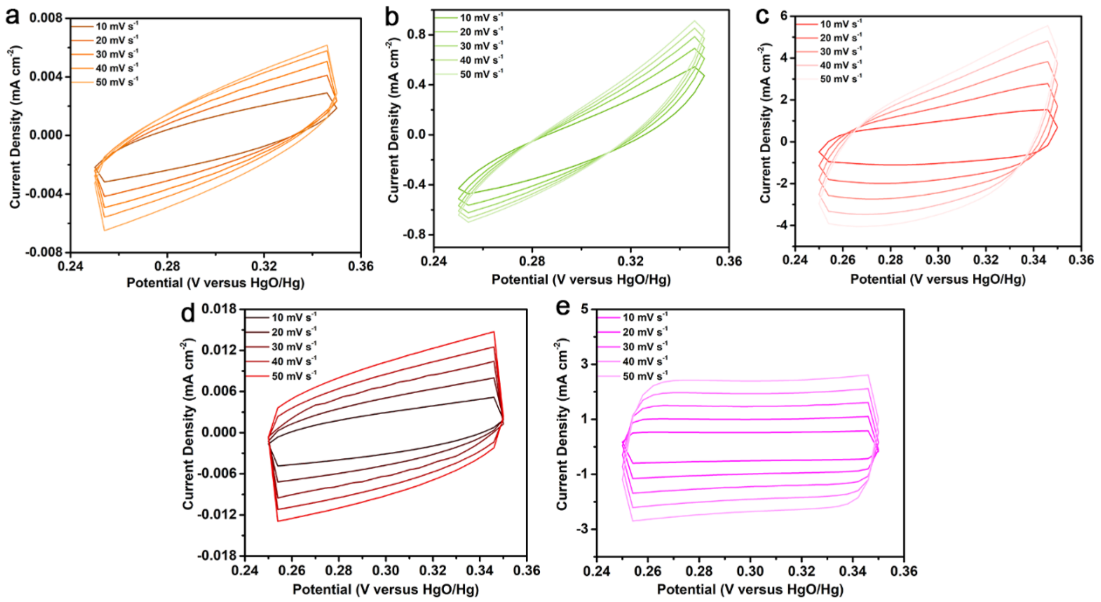

Figure S7. Electrochemical cyclic voltammetry scans recorded for a) a-Ni-MeIM, b) a-Ni-MeIM-Co-0.01, c) $Ni_{0.42}Co_{0.58}F_2$-G, d) NiF_2 and e) CoF_2. Scan rates are 10, 20, 30, 40 and 50 mV s^{-1}.

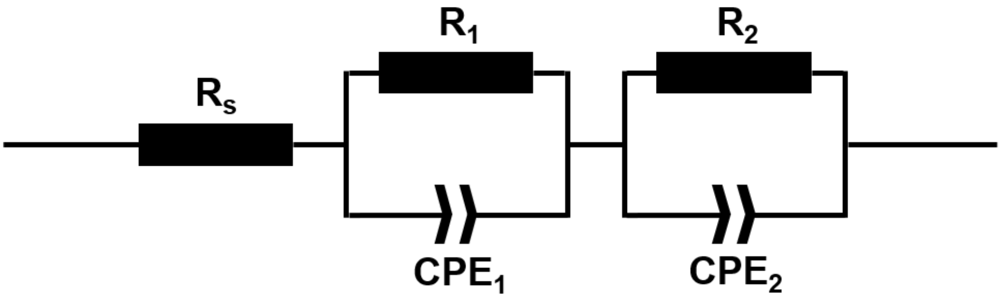

Figure S8. Equivalent circuit used for the fitting of the EIS, where R_s, R_1, R_2, CPE_1, and CPE_2 represent the solution resistance, electrode texture, charge transfer resistances and constant phase elements, respectively.

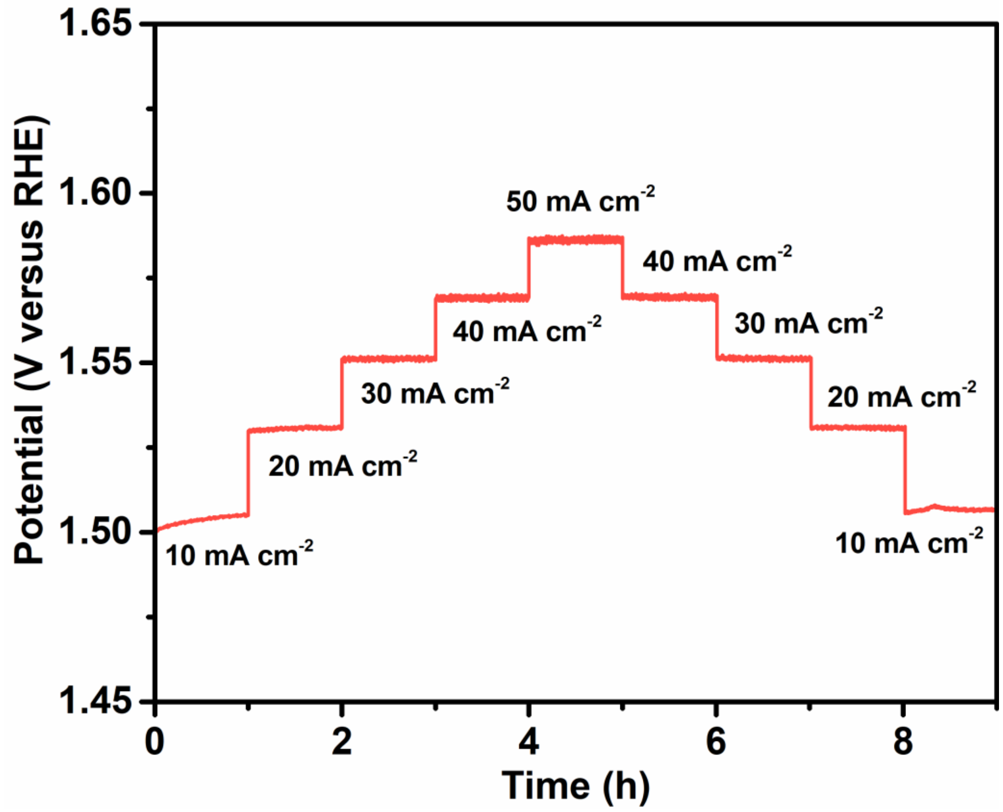

Figure S9. Chronopotentiometry test of $Ni_{0.42}Co_{0.58}F_2$-G at 10mA cm^{-2}, 20mA cm^{-2}, 30mA cm^{-2}, 40mA cm^{-2} and 50mA cm^{-2}.

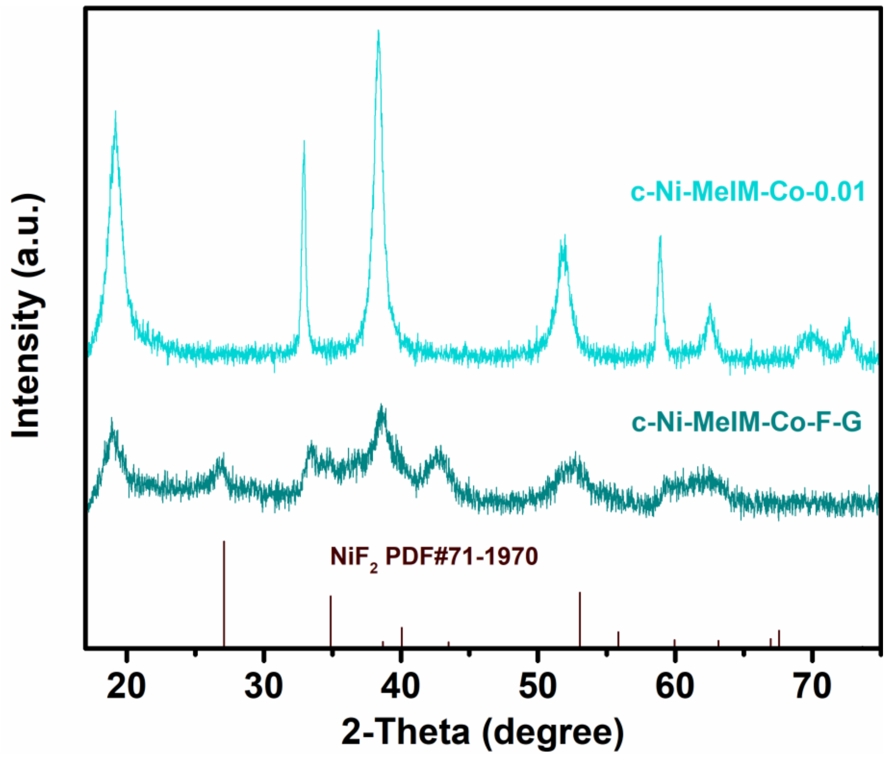

Figure S10. XRD patterns of c-Ni-MeIM-Co-0.01 and c-Ni-MeIM-Co-F-G.

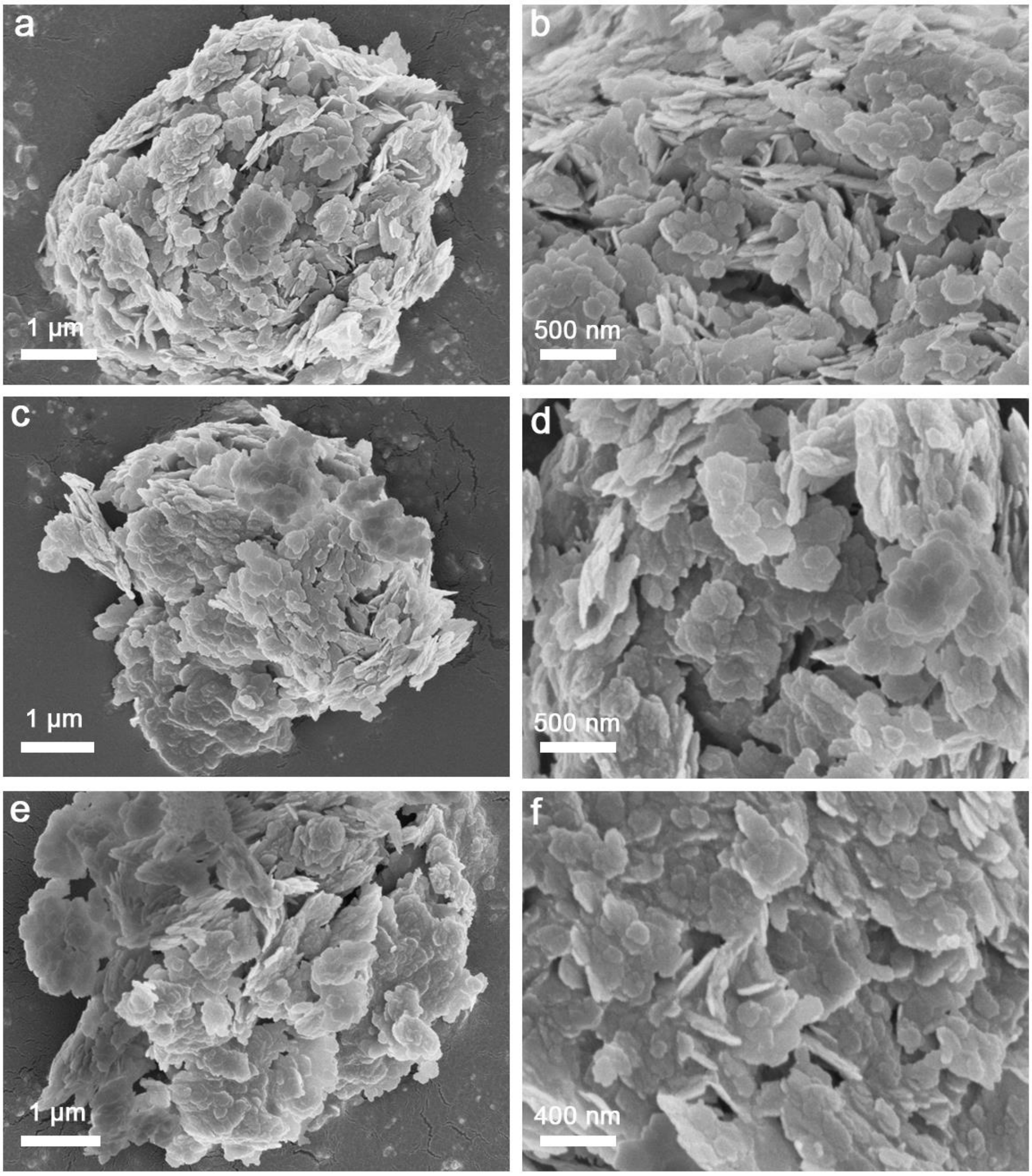

Figure S11. SEM images of a) and b) c-Ni-MeIM, c) and d) c-Ni-MeIM-Co-0.01, e) and f) c-Ni-MeIM-Co-F-G.

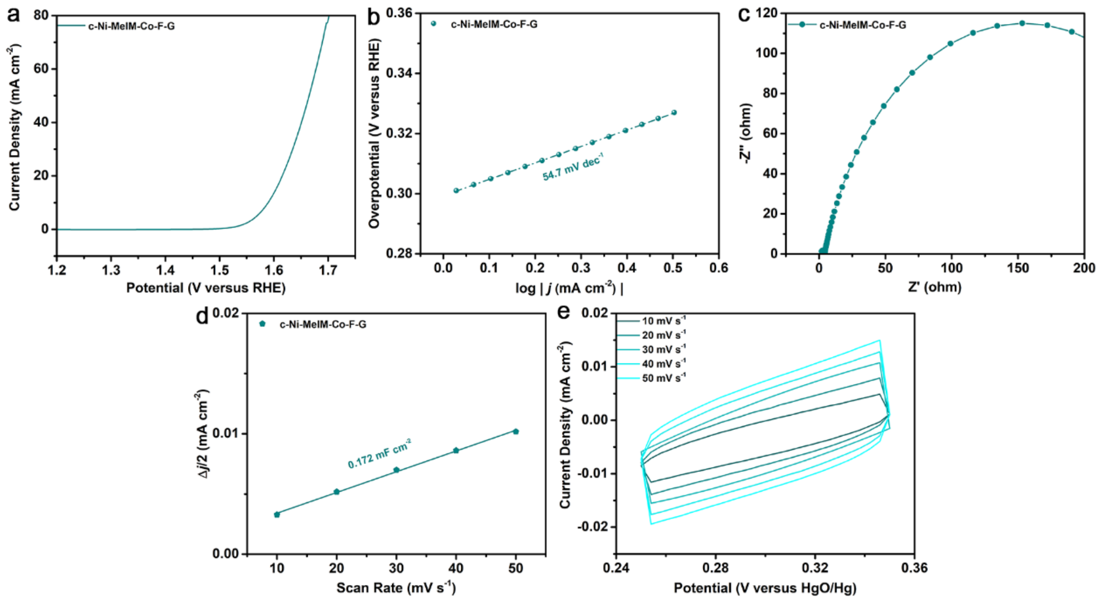

Figure S12. a) Polarization curves, b) Steady-state Tafel slope and c) Nyquist plots at 1.52 V versus RHE of c-Ni-MeIM-Co-F-G; d) The $\Delta j/2$ at 1.22 V (vs RHE) as a function of the scan rate; e) Electrochemical cyclic voltammetry scans recorded for c-Ni-MeIM-Co-F-G.

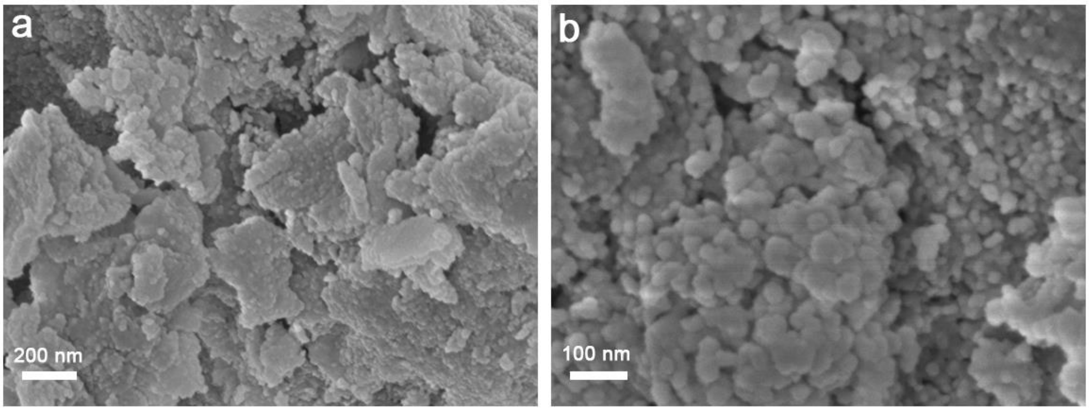

Figure S13. SEM images of $Ni_xCo_yF_2$-L.

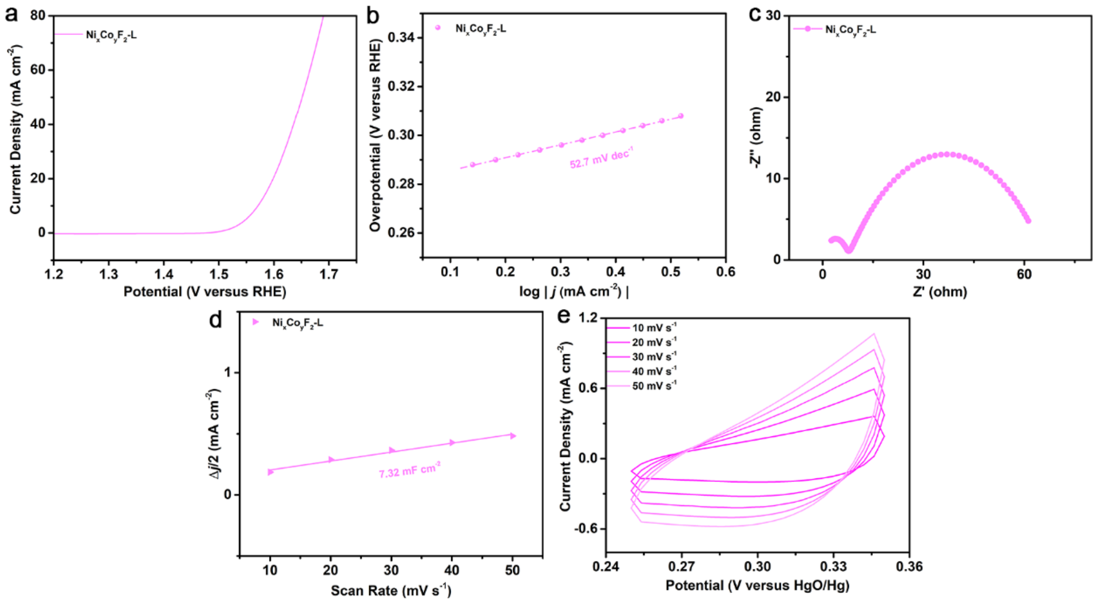

Figure S14. a) Polarization curves, b) Steady-state Tafel slope and c) Nyquist plots at 1.52 V versus RHE of $Ni_xCo_yF_2$-L; d) The Δ*j*/2 at 1.22 V (vs RHE) as a function of the scan rate; e) Electrochemical cyclic voltammetry scans recorded for $Ni_xCo_yF_2$-L.

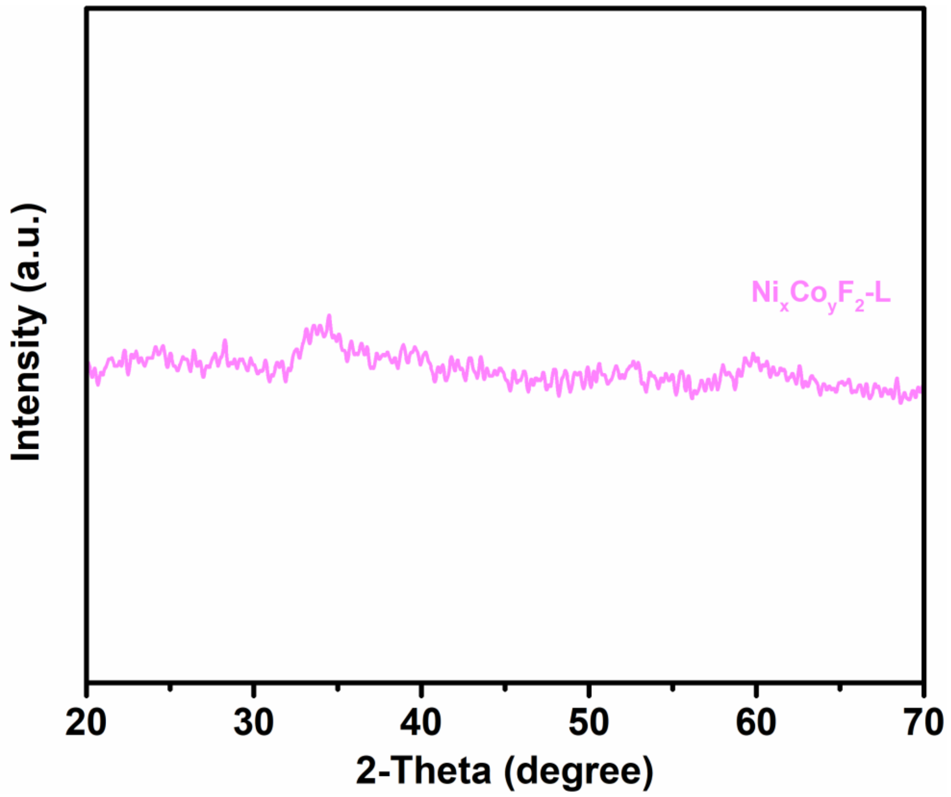

Figure S15. XRD pattern of $Ni_xCo_yF_2$-L.

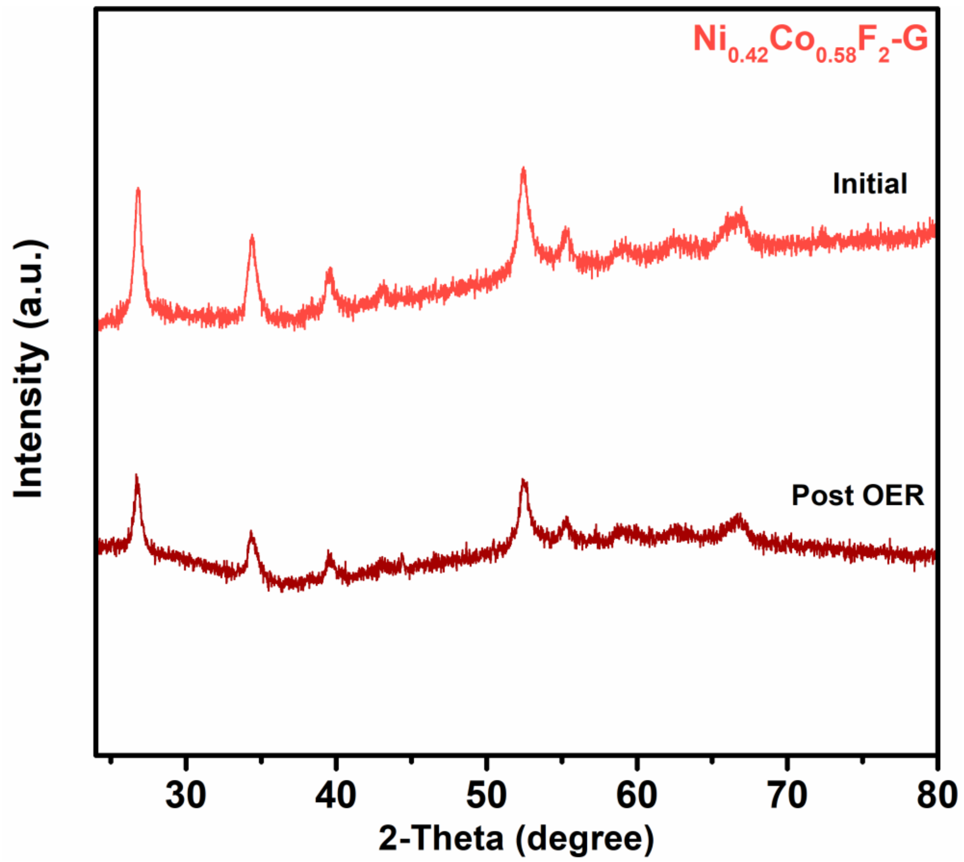

Figure S16. XRD pattern of $Ni_{0.42}Co_{0.58}F_2$-G before and after OER.

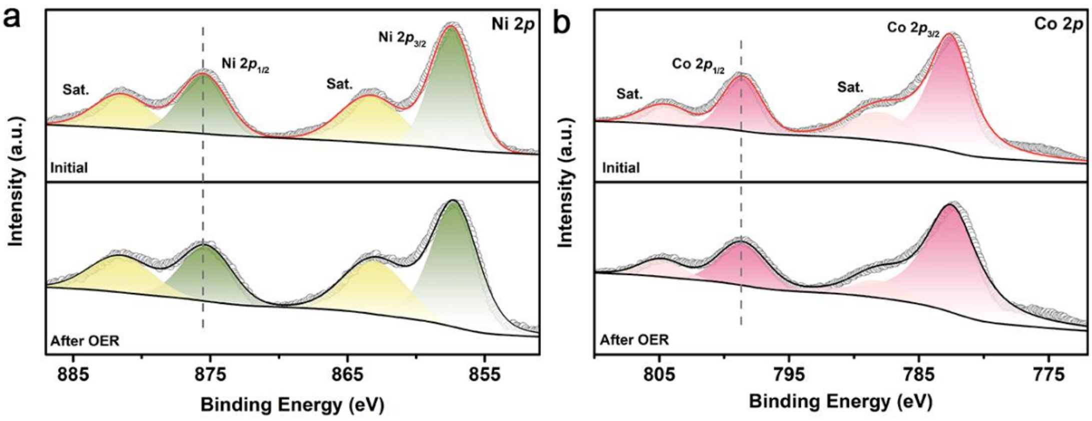

Figure S17. XPS high-resolution spectra of a) Ni 2*p* and b) Co 2*p* of $Ni_{0.42}Co_{0.58}F_2$-G before and after OER.

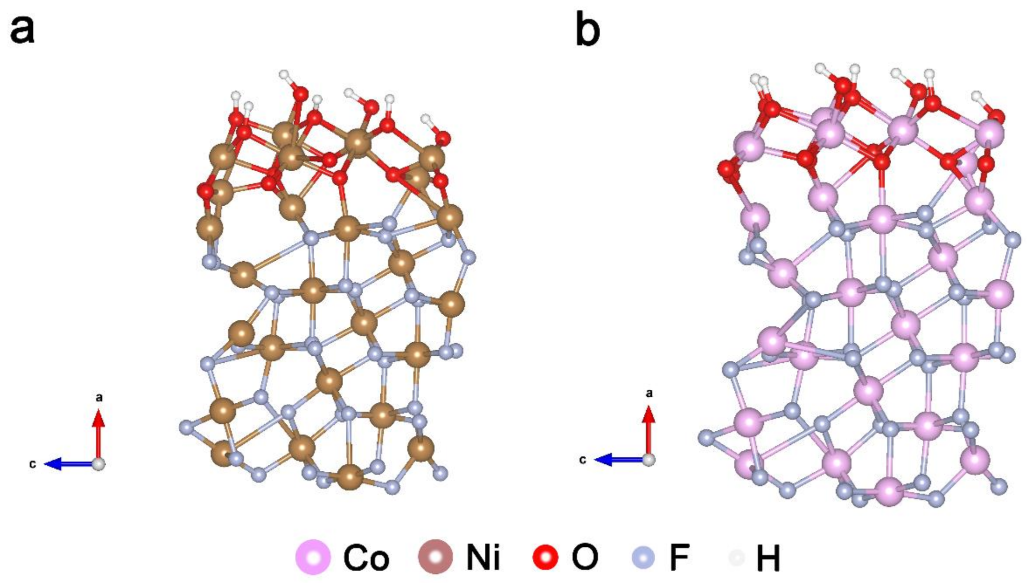

Figure S18. The crystal structure of a) $NiOOH/NiF_2$ and b) $CoOOH/CoF_2$.

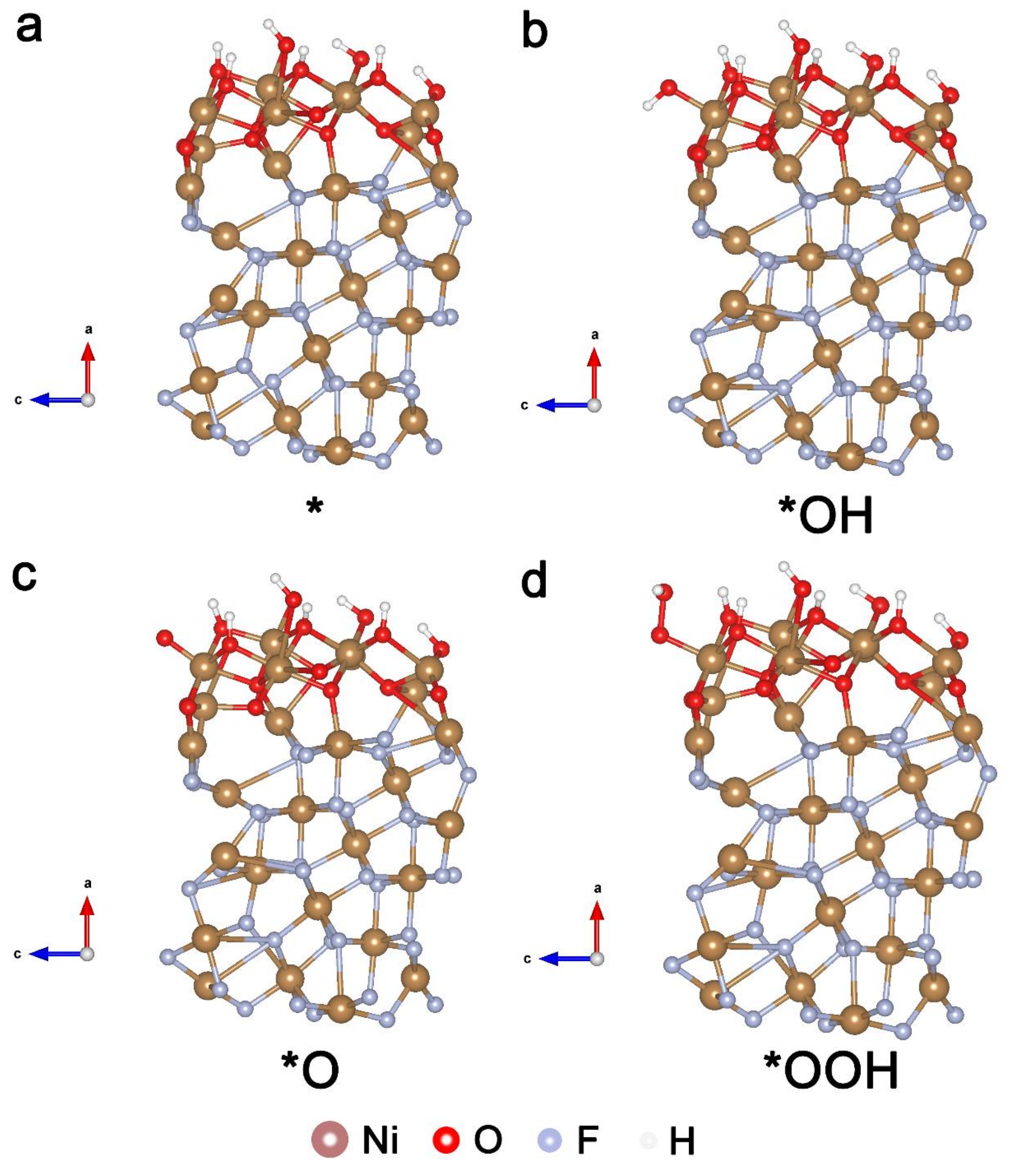

Figure S19. Reaction mechanism and model structures of intermediates involved in OER for $NiOOH/NiF_2$.

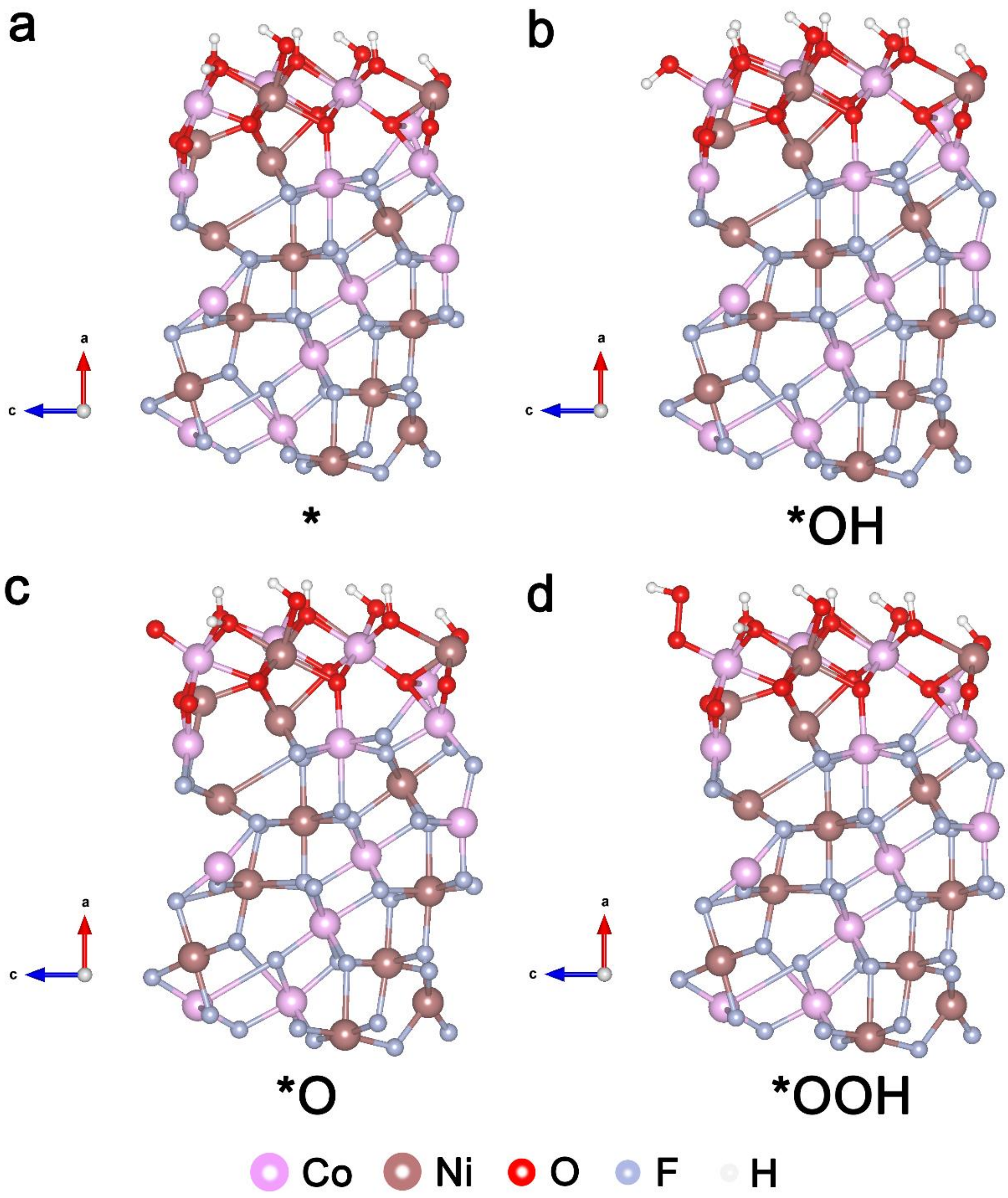

Figure S20. Reaction mechanism and model structures of intermediates involved in OER for $Ni_{0.5}Co_{0.5}OOH/NiCoF_2$.

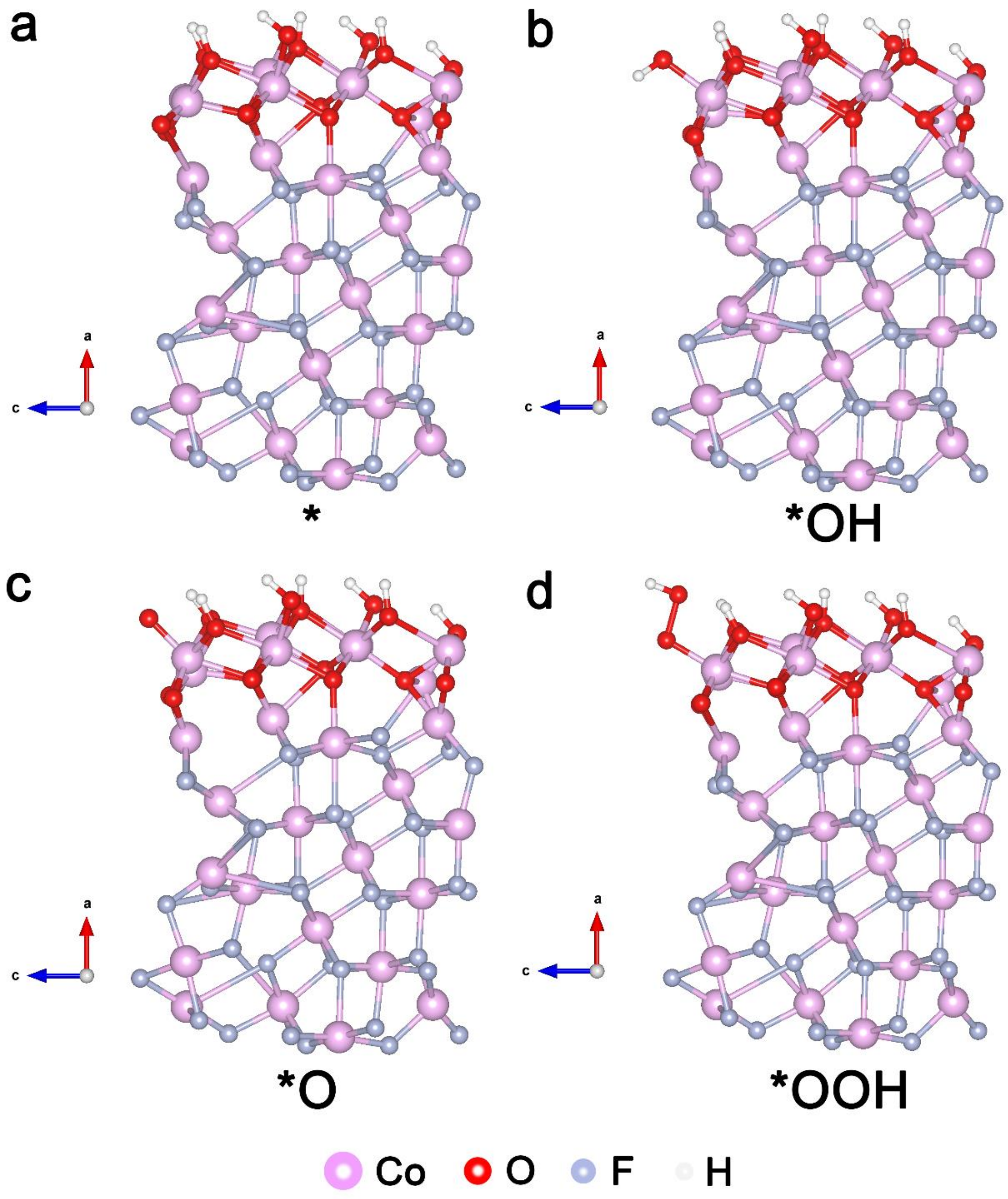

Figure S21. Reaction mechanism and model structures of intermediates involved in OER for $CoOOH/CoF_2$.

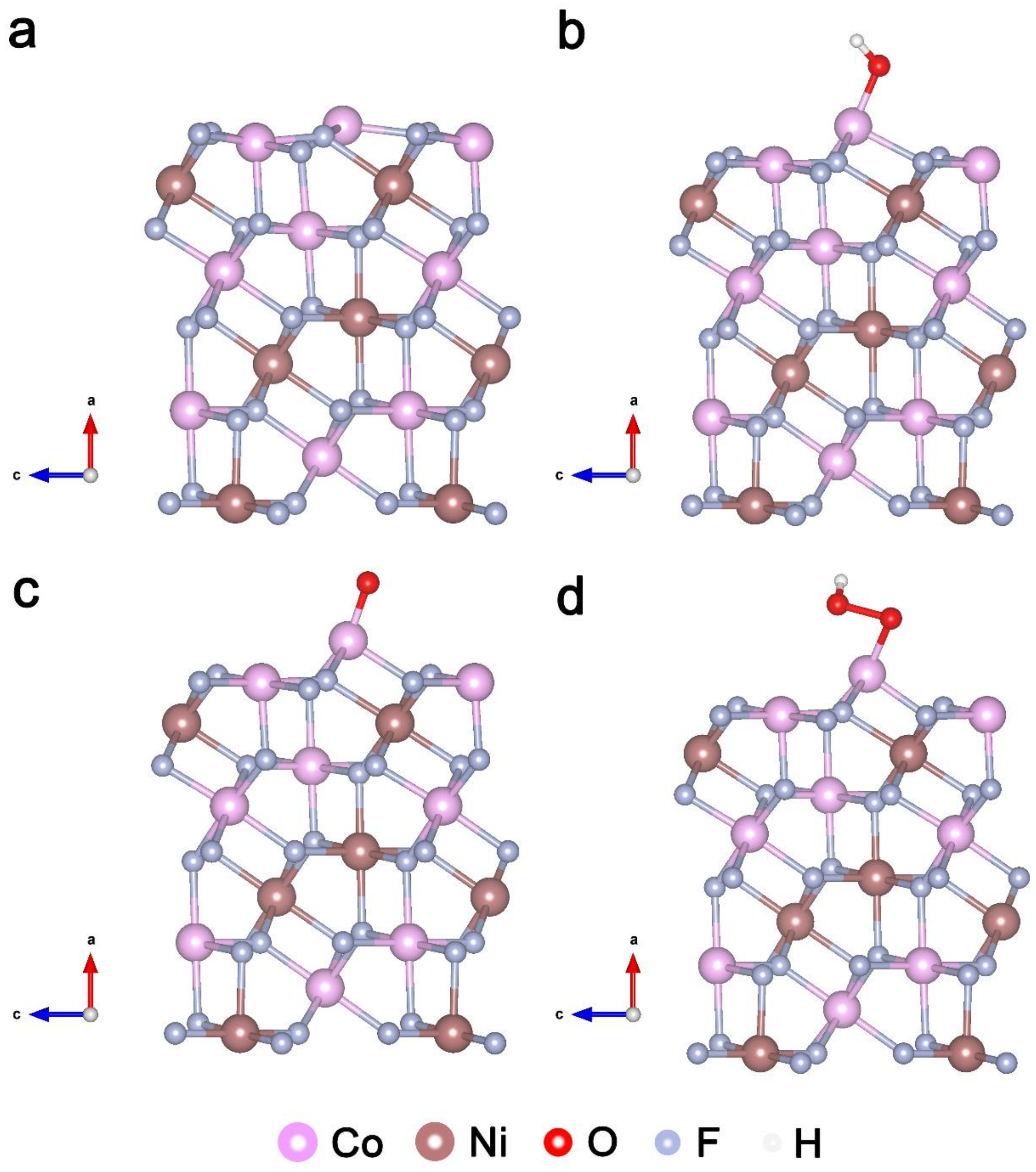

Figure S22. Reaction mechanism and model structures of intermediates involved in OER for $NiCoF_2$.

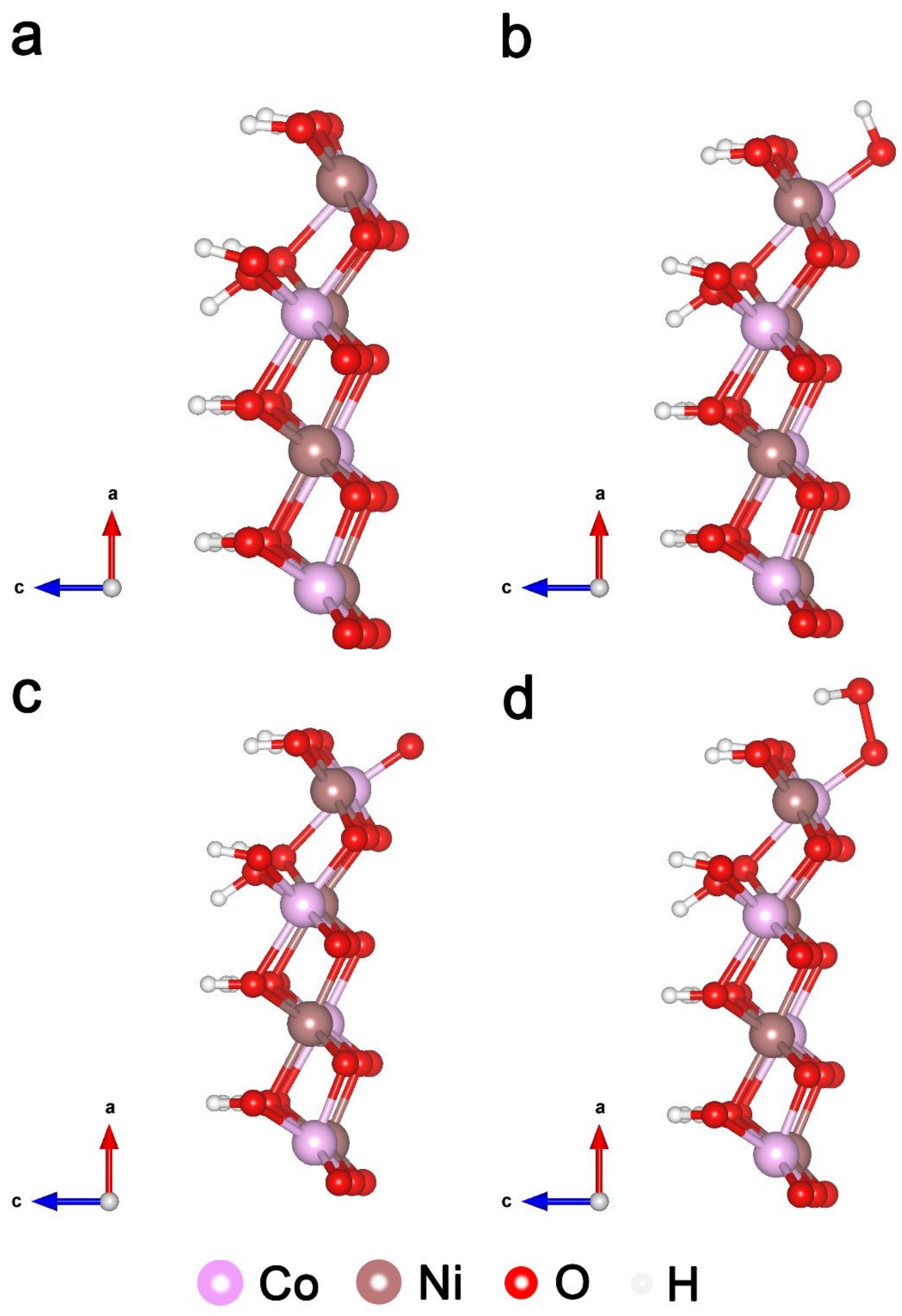

Figure S23. Reaction mechanism and model structures of intermediates involved in OER for $Ni_{0.5}Co_{0.5}OOH$.

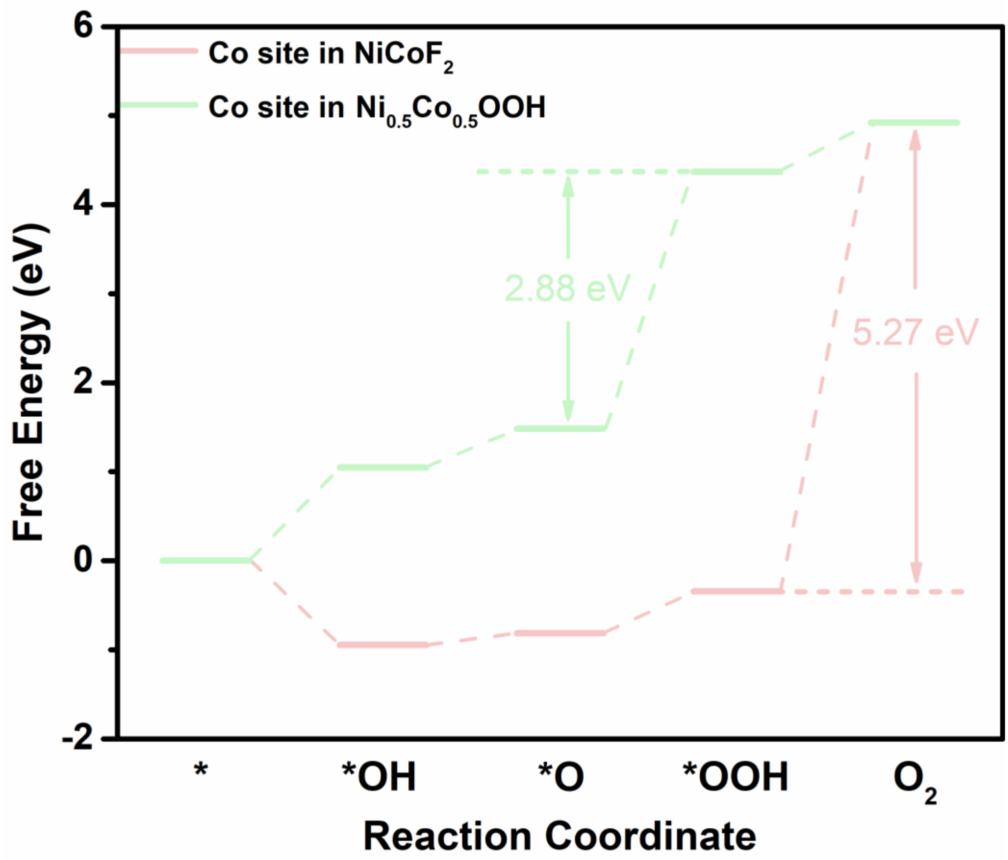

Figure S24. The calculated Gibbs free energy diagram for the four steps of OER over $Ni_{0.5}Co_{0.5}OOH$ and $NiCoF_2$.

Supplementary Tables

Table S1. Mass percentages of Ni and Co atoms in different samples by ICP-AES.

Compounds	Ni (%)	Co (%)
$Ni_{0.86}Co_{0.14}F_2$-G	38.9	6.4
$Ni_{0.42}Co_{0.58}F_2$-G	26.5	19.0
$Ni_{0.32}Co_{0.68}F_2$-G	17.1	36.9

Table S2. Concentration of Ni^{2+} and Co^{2+} in supernatant after reaction with different Co^{2+} concentrations by AAS.

Concentrations of Co^{2+} initial (mol L^{-1})	Concentrations of Ni^{2+} in supernatant (mol L^{-1})	Concentrations of Co^{2+} in supernatant (mol L^{-1})
0	0.0003	0
0.002	0.0006	0.0008
0.01	0.0017	0.0056
0.04	0.0037	0.0308

Table S3. Comparisons of the Tafel slopes and overpotentials at the current density of 10 mA cm-2 of reported NiCo-based catalysts for OER in 1 M KOH.

Catalysts	Overpotential (mV)	Tafel slope (mV dec^{-1})	Reference
$Co(OH)_2$@Ni	330	100	[1]
Co–Ni_3C/Ni@C	325	67.76	[2]
$Ni_{0.25}Co_{0.75}(OH)_2$	352	72.0	[3]
$Co(OH)_2$@$Ni(OH)_2$/CC	330	223	[4]
Co–Ni (trace)/NCNTs	337	94	[5]
Ni-Co LDH@MQDs	316	79	[6]
$Ni_{0.25}Co_{0.65}S_{0.4}Se_{0.6}$	358	64	[7]
$Ni_{0.13}Co_{0.87}S_{1.097}$	316	54.72	[8]
KNCF82	310	49	[9]
$Ni_{0.6}CoP$	300	80	[10]
NiCo-2.0-800HP	320	84	[11]
CNTs@NiCoP/C	297	57.35	[12]
$Co_{1.4}Ni_{0.6}O_2$	366.3	113.6	[13]
Ru-Ni-Co-P/NC	318	84	[14]
MIL-88@CoNi-LDHs	314	45.55	[15]
$Ni_{0.42}Co_{0.58}F_2$-G	313	39	This work

Supplementary References

1. A. Roy, M. Z. Tariq, M. La, D. Choi and S. J. Park, *Journal of Electroanalytical Chemistry*, 2022, **920**.
2. X. Jia, M. Wang, G. Liu, Y. Wang, J. Yang and J. Li, *International Journal of Hydrogen Energy*, 2019, **44**, 24572-24579.
3. Y. Wang, C. Yang, Y. Huang, Z. Li, Z. Liang and G. Cao, *Journal of Materials Chemistry A*, 2020, **8**, 6699-6708.
4. Y. Wang, Y. He and M. Zhou, *Applied Surface Science*, 2019, **479**, 1270-1276.
5. Y. Shi, J. Cai, X. Zhang, Z. Li and S. Lin, *International Journal of Hydrogen Energy*, 2022, **47**, 7761-7769.
6. L. Song, X. Zhang, X. Du, S. Zhu, Y. Xu and Y. Wang, *Phys Chem Chem Phys*, 2022, **24**, 24902-24909.
7. M. Wang, Z. Dang, M. Prato, D. V. Shinde, L. De Trizio and L. Manna, *ACS Applied Nano Materials*, 2018, **1**, 5753-5762.
8. J. Zhang, D. Zhang, R. Zhang, N. Zhang, C. Cui, J. Zhang, B. Jiang, B. Yuan, T. Wang, H. Xie and Q. Li, *ACS Applied Energy Materials*, 2018, **1**, 495-502.
9. S. Guddehalli Chandrappa, P. Moni, D. Chen, G. Karkera, K. R. Prakasha, R. A. Caruso and A. S. Prakash, *ACS Applied Energy Materials*, 2021, **4**, 13425-13430.
10. B. Qiu, L. Cai, Y. Wang, Z. Lin, Y. Zuo, M. Wang and Y. Chai, *Advanced Functional Materials*, 2018, **28**.
11. N. L. W. Septiani, Y. V. Kaneti, K. B. Fathoni, Y. Guo, Y. Ide, B. Yuliarto, X. Jiang, Nugraha, H. K. Dipojono, D. Golberg and Y. Yamauchi, *Journal of Materials Chemistry A*, 2020, **8**, 3035-3047.
12. Y. Zhao, G. Fan, L. Yang, Y. Lin and F. Li, *Nanoscale*, 2018, **10**, 13555-13564.
13. H. Li, L. Chen, P. Jin, H. Lv, H. Fu, C. Fan, S. Peng, G. Wang, J. Hou, F. Yu and Y. Shi, *Dalton Trans*, 2020, **49**, 6587-6595.
14. D. Wang, L. Gu, X. Luo, R. Su, Y. Shang, Y. Wang, S. Hao and Y. Yang, *Journal of Electroanalytical Chemistry*, 2022, **924**.
15. D. Han, L. Hao, M. Chang, J. Dong, Y. Gao and Y. Zhang, *J Colloid Interface Sci*, 2022, **634**, 14-21.
